# Prospective genomic surveillance of methicillin-resistant *Staphylococcus aureus* (MRSA) associated with bloodstream infection, England, 1 October 2012 to 30 September 2013

**DOI:** 10.2807/1560-7917.ES.2019.24.4.1800215

**Published:** 2019-01-24

**Authors:** Michelle S Toleman, Sandra Reuter, Dorota Jamrozy, Hayley J Wilson, Beth Blane, Ewan M Harrison, Francesc Coll, Russell J Hope, Angela Kearns, Julian Parkhill, Sharon J Peacock, M Estée Török

**Affiliations:** 1 University of Cambridge, Department of Medicine, Cambridge, United Kingdom; 2 Wellcome Sanger Institute, Hinxton, United Kingdom; 3 Cambridge University Hospitals NHS Foundation Trust, Cambridge, United Kingdom; 4 University of Freiburg, Institute for Infection Prevention and Hospital Epidemiology, Freiburg, Germany; 5 London School of Hygiene and Tropical Medicine, London, United Kingdom; 6 Public Health England, National Infection Service, Colindale, London, United Kingdom; 7 Public Health England, Clinical Microbiology and Public Health Laboratory, Cambridge, United Kingdom

**Keywords:** Methicillin-resistant Staphylococcus aureus, MRSA, bacteraemia, surveillance, epidemiology, public health, whole-genome sequencing, WGS

## Abstract

**Background:**

Mandatory reporting of methicillin-resistant *Staphylococcus aureus* (MRSA) bloodstream infections (BSI) has occurred in England for over 15years. Epidemiological information is recorded, but routine collection of isolates for characterisation has not been routinely undertaken. Ongoing developments in whole-genome sequencing (WGS) have demonstrated its value in outbreak investigations and for determining the spread of antimicrobial resistance and bacterial population structure. Benefits of adding genomics to routine epidemiological MRSA surveillance are unknown.

**Aim:**

To determine feasibility and potential utility of adding genomics to epidemiological surveillance of MRSA.

**Methods:**

We conducted an epidemiological and genomic survey of MRSA BSI in England over a 1-year period (1 October 2012­–30 September 2013).

**Results:**

During the study period, 903 cases of MRSA BSI were reported; 425 isolates were available for sequencing of which, 276 (65%) were clonal complex (CC) 22. Addition of 64 MRSA genomes from published outbreak investigations showed that the study genomes could provide context for outbreak isolates and supported cluster identification. Comparison to other MRSA genome collections demonstrated variation in clonal diversity achieved through different sampling strategies and identified potentially high-risk clones e.g. USA300 and local expansion of CC5 MRSA in South West England.

**Conclusions:**

We demonstrate the potential utility of combined epidemiological and genomic MRSA BSI surveillance to determine the national population structure of MRSA, contextualise previous MRSA outbreaks, and detect potentially high-risk lineages. These findings support the integration of epidemiological and genomic surveillance for MRSA BSI as a step towards a comprehensive surveillance programme in England.

## Introduction

In 2001, faced with increasingly high rates of methicillin-resistant S*taphylococcus aureus* (MRSA) bloodstream infections (BSI) at the turn of the century, the United Kingdom (UK) Department of Health mandated surveillance of MRSA BSI in England. This was followed in 2005 by enhanced surveillance to collect clinical and epidemiological information [1,2]. A number of infection prevention and control (IPC) measures were also introduced, such as strengthened antimicrobial stewardship, MRSA screening with decolonisation of all emergency hospital admissions [3] and use of care bundles for patients with intravascular catheters and indwelling urinary catheters [4]. Over the past decade, there has been a remarkable decline in the incidence of MRSA BSI in England [1,2]. Surveillance and IPC interventions are likely to have played a major role in this success, although it is unclear whether potential changes in the epidemiology of MRSA may also have contributed [5,6].

The national MRSA BSI surveillance programme conducted by Public Health England (PHE) did not include routine submission of isolates for characterisation. Isolates submitted to the PHE Staphylococcal Reference Service were highly selected and were submitted in order to type isolates for the investigation of suspected nosocomial and community outbreaks, for selected sentinel surveillance programmes and/or to detect specific genes in isolates from patients with suspected toxin-mediated disease. Strain characterisation was undertaken using staphylococcal protein A (*spa*-) typing, multilocus sequence typing (MLST), SCC*mec*-subtyping and toxin gene profiling. It is possible that a large amount of information regarding the population of disease-causing MRSA in England may have been missed as a result of this *ad hoc *approach.

Microbial WGS provides increased discriminatory power to resolve outbreaks and identify emerging MRSA lineages compared with conventional typing methods [7]. WGS has been used to investigate MRSA outbreaks in the UK [8-12] and to examine the population genetic structure of MRSA in the UK and globally [13,14]. These studies have largely been conducted through grant-funded academic research, rather than by public health programmes, using isolates from surveillance programmes such as the British Society of Antimicrobial Chemotherapy (BSAC) Antibiotic Resistance Surveillance Programme [13] or the European Antimicrobial Resistance Surveillance Network, EARS-Net [15]. Both programmes systematically collect a subset of bloodstream isolates from sentinel laboratories and routinely undertake phenotypic typing methods. Both programmes were established to monitor antimicrobial resistance, before the widespread use of WGS.

Combined with comprehensive, systematic sampling regimens WGS technologies now provide the opportunity to study the natural history of successful MRSA clones at great resolution and to identify clonal expansions to monitor in case of widespread dissemination [16]. National BSI surveillance was originally introduced in England to compare MRSA rates between hospitals and later enhanced to aid direction of clinical interventions [2]. We conducted a proof-of-principle study to determine the feasibility and potential benefits of combining prospective epidemiological and genomic surveillance of MRSA BSI on a national scale within a public health organisation. We aimed to determine what information could be gathered by combining epidemiological surveillance and routine whole-genome sequencing of isolates and to identify the potential obstacles to implementation of this strategy.

## Methods

### Study design, setting and participants

We conducted a prospective, observational cohort study of all cases of MRSA BSI in England from 1 October 2012 to 30 September 2013. Cases were defined as those patients reported to PHE as having a blood culture positive for MRSA by the submitting laboratory. At the time of the study the population of England, served by PHE, was approximately 53.4 million.

### Data sources

In accordance with national policy, epidemiological and microbiological data on MRSA BSI cases is submitted electronically to the mandatory enhanced surveillance scheme (MESS) by infection control teams in acute National Health Service (NHS) Trusts. Mandatory data variables included patient demographics, details of hospital admission, date of BSI and location of acquisition (community or hospital). Epidemiological and microbiological data of cases with BSI during the study period was extracted from this database for use in this study. PHE reference laboratory test results were initially linked with demographic, clinical and geographic information from the MESS and then anonymised by PHE staff. Anonymised data were subsequently linked to DNA sequence data by University of Cambridge staff.

### Isolate collection and laboratory testing

During the study period, all NHS diagnostic microbiology laboratories in England were invited to submit MRSA bloodstream isolates to the Staphylococcal Reference Laboratory, PHE Colindale, for characterisation. Isolates were cultured on nutrient agar and underwent *spa*-typing [17] and multiplex PCR to confirm species identification and determination of the *mecA* and *luk-PV* status [18]. Isolates were stored at -80 °C using Microbank cryovials (Pro-Laboratory Diagnostics, Cheshire, UK) pending further analyses.

### DNA extraction and whole genome sequencing

Isolates were retrieved from storage, sub-cultured onto nutrient agar slopes, and transferred to the Department of Medicine at the University of Cambridge. Each sample was cultured onto Columbia Blood Agar (Oxoid, Basingstoke, UK) and identified using a commercial latex agglutination kit (Pastorex Staph Plus, Bio Rad Laboratories, Hemel Hempstead, UK). Antimicrobial susceptibility testing was performed using the Vitek-2 system (bioMérieux, Marcy l’Etoile, France). DNA was extracted, libraries prepared, and 150-bp paired-end sequences determined on an Illumina HiSeq2000 as previously described [19]. Phylogenetic trees were visualised using FigTree (http://tree.bio.ed.ac.uk/software/figtree/) and iTOL (http://itol.embl.de/).

### Ethical statement

Written informed consent was not required for this study as data and isolates were collected as part of national surveillance programme for MRSA bloodstream infections, which is exempt from this requirement.

The study protocol was approved by the National Research Ethics Service (ref: 11/EE/0499), and by the Cambridge University Hospitals NHS Foundation Trust R&D Department (ref: A092428).

### Genomic analysis

Genomes were assembled using an assembly and improvement pipeline [20]. MLST sequence types (STs) were assigned from the sequence data [21] (https://github.com/sanger-pathogens/mlst_check) and STs were assigned to clonal complexes (CC). Sequence data were mapped using SMALT (http://www.sanger.ac.uk/science/tools/smalt-0) to the reference genome for particular CCs (CC5, N315, GenBank *accession* number BA000018; CC8, FPR3757, GenBank *accession* number CP000255; CC22, EMRSA15, GenBank *accession* number HE681097). The core genome alignment excluding mobile genetic elements, indels and repetitive regions was generated for each CC and was used in phylogenetic estimates using RAxML with 100 bootstraps [22].

Isolates were *spa* genotyped using *in-silico* PCR to extract the *spa* gene X region from assembled genomes using previously described primers [23]. The *spa*-type was determined using an online *spa*-typer tool (http://spatyper.fortinbras.us/). The types generated through s*pa*-genotype and laboratory determined *spa*-typing methods were compared to determine concordance.

Bacterial DNA sequences were deposited in the European Nucleotide Archive (ENA), (https://www.ebi.ac.uk/ena), under study number ERP005128. Accession numbers, details of reads, depth of coverage and N50 are provided in Supplementary Table S1. For subsequent analyses we sourced MRSA sequence data from previously published studies. These included: (i) a prospective observational cohort study of all MRSA carriage and clinical isolates submitted and processed in Cambridge University Hospital NHS Foundation Trust, Cambridgeshire, UK between 2012 and 2013 [24], (ii) MRSA bloodstream isolates collected by the BSAC BSI Surveillance Programme between 2001 and 2010 [13], (iii) USA300 isolates collected in New York, United States (US) between 2009 and 2011 [25], (iv) MRSA isolates from outbreak investigations at a UK hospital [9,10,12].

## Results

A total of 903 MRSA BSI cases were reported to MESS during the study period (Supplementary Figure S1). Gender was recorded for 98% of cases and 584 (65%) of cases were male. Age was recorded for all but two cases, with a median age of 72 years (range 0–103 years; interquartile range (IQR) 56–84 years). A total of 111 laboratories participated in the study.

A total of 559 MRSA bloodstream isolates were received. Following quality control procedures 134 isolates were excluded, and 425 isolates were included in the analysis. The reasons of exclusion were as follows: duplicate isolates (n = 50); not MRSA (n = 15); inadequate isolate growth (n = 2); isolates collected outside of the study dates (n = 16); isolates submitted in error (n = 3); non-bloodstream isolates (n = 2); isolates from Wales (n = 28); and isolates from Northern Ireland (n = 18).

**Figure 1:**
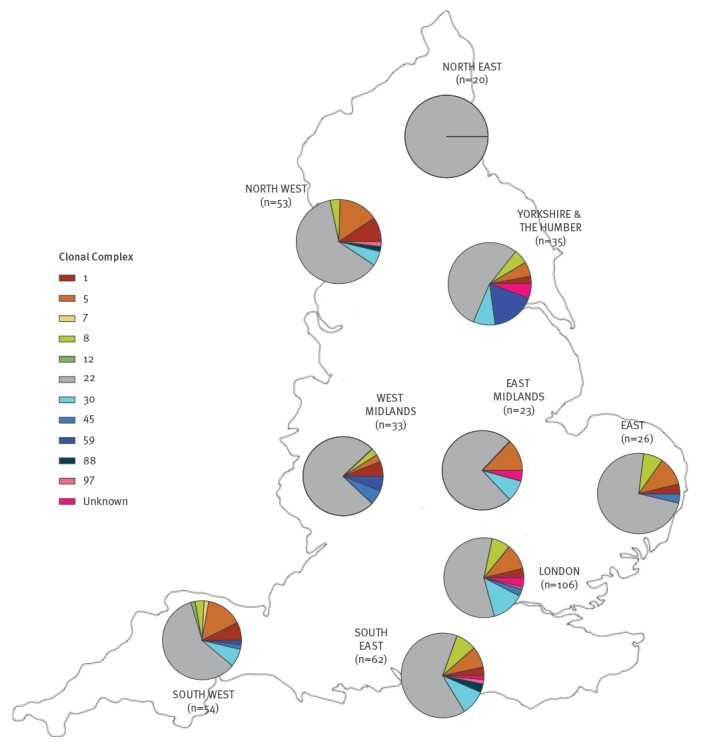
Map with breakdown of the proportions of each CC within the sequenced PHE bloodstream infection isolate collection from submitting regions, England, 1 October 2012–30 September 2013 (n = 425)

CC: clonal complex; PHE: Public Health England.

Of 903 reported BSI cases occurring in England during the study period, 47% (n = 425) had isolates that were sequenced and analysed (Figure 1). All of the 425 sequenced isolates were *mecA* positive by laboratory-PCR. PCR testing identified 8.7% (n = 37) of the isolates as PVL-positive. Based on sequence data, 65% (n = 276) were assigned to CC22. Other CCs were represented at lower frequencies: CC5 n = 42; CC30 n = 33; CC8 n = 22; CC1 n = 19; CC59 n = 9; CC45 n = 7; other/unknown CCs n = 17. The number of isolates and variation in the CCs isolated from each region is shown in Figure 1. No associations were found between particular CCs and community vs hospital onset (Supplementary Table S2).

The number of isolates received by Public Health England from each of the labelled regions is indicated in brackets. Thirteen isolates did not have a region assigned.

### Comparison of blood stream infection surveillance and universal methicillin-resistant *Staphylococcus aureus* sampling

**Figure 2:**
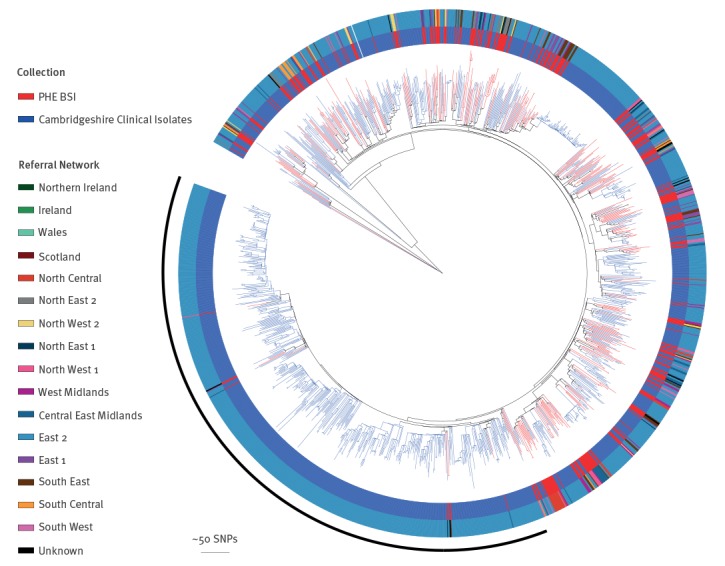
Phylogenetic tree comparing CC 22 PHE BSI isolates, England, October 2012-September 2013 (n = 276), to a single isolate per patient from the previously published universal sample collection from Cambridgeshire, England, April 2012–April 2013 (n = 1035)

BSI: Blood stream infections; CC: clonal complex; PHE: Public Health England; SNPs: single nucleotide polymorphisms.

We compared the most common clone in our collection, CC22 (n = 276), with CC22 genomes generated by a prospective study that sequenced MRSA isolates from every positive case (carriage and clinical samples) identified at a single diagnostic microbiology laboratory that processed samples from three hospitals and 75 general practitioner (GP) surgeries in Cambridgeshire between April 2012 and April 2013 [24]. This Cambridgeshire collection was used to represent the diversity of carriage and clinical isolates within a defined geographical area, as a national collection of carriage and clinical isolates was not available. A phylogeny was constructed for the genomes from the national BSI collection within this Cambridgeshire collection (Figure 2), in order to determine whether those isolates causing BSI were clonally related, or distributed throughout the phylogenetic tree.

Public Health England bloodstream infection CC22 isolates (n = 276) are indicated by red branches. The first isolate from each patient from the previously published universal sample collection from Cambridgeshire (n = 1,035) are indicated by the blue branches [24].

Mid-point rooted maximum likelihood tree based on SNPs in the core genome alignment generated after mapping against the reference genome EMRSA-15. The inner ring denotes the collection and the outer represents referral network of submitting laboratory. The arc indicates a large expansion in the Cambridgeshire region, which is underrepresented in the BSI only surveillance.

As shown in Figure 2, isolates from our national MRSA BSI collection were dispersed throughout the Cambridgeshire phylogeny, ruling out any association between a particular lineage and BSI. Comparing the national BSI collection to WGS of universal sampling in Cambridgeshire also demonstrates that some lineages are under-represented when undertaking BSI-based (rather than clinical/carriage based) surveillance. For example, a large expansion (indicated with an arc on the figure) was seen in the Cambridgeshire phylogeny, with only eight of the Cambridgeshire isolates within the national MRSA BSI collection from the East of England.

**Figure 3:**
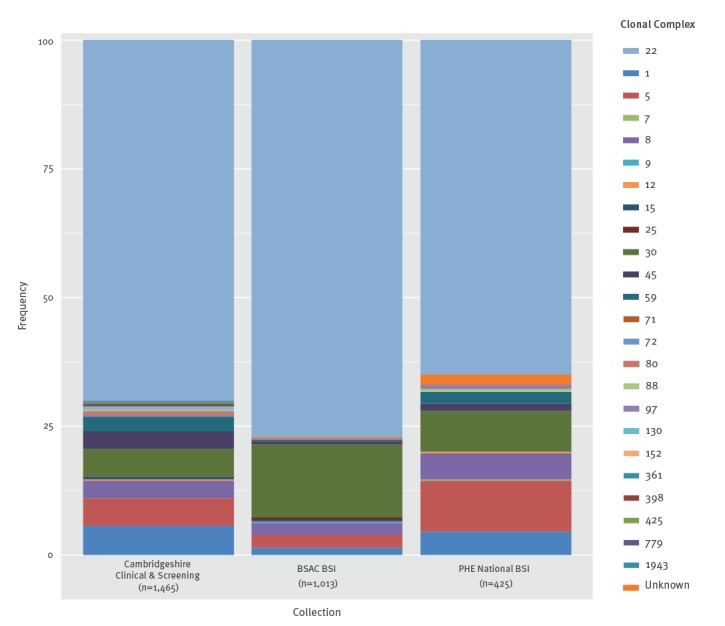
Diversity of lineages (CC) within three isolate collections: Carriage and clinical samples from the Cambridgeshire study of MRSA^a^; the national PHE BSI collection^b^ and a national BSAC BSI collection^c^

BSAC: British Society of Antimicrobial Chemotherapy; BSI: blood stream infections; CC: clonal complex; MRSA: methicillin-resistant *Staphylococcus aureus*. PHE: Public Health England.

^a^ Carriage and clinical samples from a 1-year-long Cambridgeshire study of MRSA (April 2012-April 2013) [24].

^b ^The national PHE BSI collection (October 2012-October 2013).

^c^ National BSAC BSI collection (2000-2010) [13].

To explore the effect of different sampling strategies on MRSA lineage diversity we conducted a comparison of CCs within three different MRSA collections: this national MRSA BSI collection (October 2012–October 2013), isolates from the Cambridgeshire study (April 2012–April 2013) [24], and MRSA BSI isolates from the British Society of Antimicrobial Chemotherapy (BSAC) BSI Surveillance Programme from 2000–2010 [13] (Figure 3). Despite the different sampling strategies and time frames, we found that CC22 was the dominant lineage in all collections. Both of the BSI-based collections showed a lower diversity of lineages than seen in the 1-year Cambridgeshire study. Furthermore, the BSAC collection, which collected BSI from up to 40 laboratories in the UK between 2001 and 2010, showed the most limited diversity. This may have resulted from a decline in certain lineages e.g. EMRSA-16 (CC30) during the 10-year collection period.

### Contextualisation of previously recognised outbreaks

**Figure 4:**
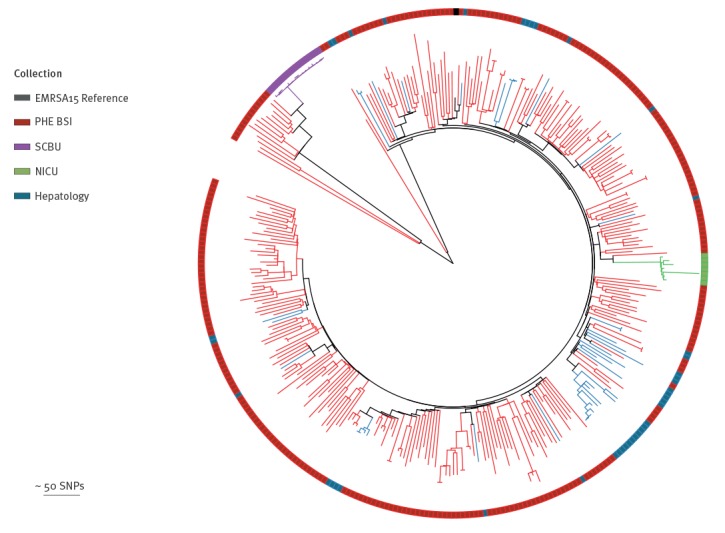
Phylogenetic tree showing CC 22 isolates from the PHE BSI collection, England, October 2012–September 2013 (n = 276) providing contextualisation of previously published outbreaks at Cambridge University Hospitals, England (n = 64)

BSI: bloodstream infections; NICU: neonatal intensive care unit; PHE: Public Health England; SCBU: special care baby unit; SNP: single nucleotide polymorphisms.

Reuter *et al.* have previously demonstrated that it is possible to use sequence data from BSAC MRSA BSI collection (2001–2010) to provide genomic context for local MRSA outbreaks within a single hospital setting [13]. We conducted a similar analysis, using the national MRSA BSI collection as context, to see if this might be feasible using a smaller sample of BSI collected during the study period of 1-year. We found that previous outbreaks in a neonatal intensive care unit [10] and a paediatric intensive care unit [9] were easily identifiable as discrete clusters, as shown in Figure 4. Furthermore, MRSA isolates from a suspected outbreak on a hepatology ward [12] were scattered throughout the phylogeny, refuting the outbreak as had been shown previously.

Mid-point rooted maximum likelihood tree based on SNPs in the core genome alignment generated after mapping against the reference genome EMRSA-15. Isolates from NICU (n = 7) are indicated in green [10]. Isolates from an outbreak on a SCBU which extended into epidemiologically linked cases in the community (n = 15) are indicated in purple [9]. Isolates from a suspected but disproven outbreak on a hepatology ward (n = 42) are indicated in blue [12].

### Monitoring and detection of emerging or high-risk lineages

**Figure 5:**
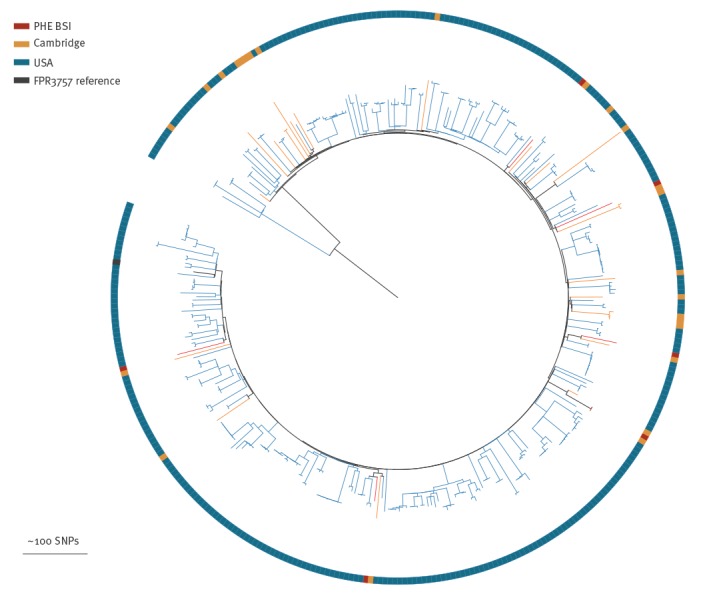
Phylogenetic tree showing USA300 isolates from the PHE BSI collection, alongside previously published USA300 isolates from a universal sample collection in Cambridgeshire and from the United States

PHE: Public Health England; SNP: single nucleotide polymorphisms; USA: United States of America.

One key aim of a national MRSA surveillance is the identification and monitoring of emerging and/or high-risk MRSA lineages. One such lineage is the USA300 lineage, which was first identified in 1999 and has subsequently caused an epidemic of skin and soft tissue infection (SSTI) in the US [26,27]. The widespread dissemination of USA300 in otherwise healthy people and its spread into hospitals has made this a high-risk strain. However, despite multiple introductions into a number of countries, genomic surveillance has shown that to date, minimal transmission of USA300 has occurred in Europe [28-32]. We examined the national MRSA BSI collection and found that six of the 22 CC8 isolates were phylogenetically defined as USA300 and were widely dispersed throughout the collection, indicating multiple introductions of USA300 into England (Figure 5). Given the observation that USA300 is commonly associated with SSTI (which are rarely sampled), and the limitations of BSI-based sampling, it is likely that the prevalence of USA300 in the UK may be higher than detected in this study.

PHE BSI USA300 isolates (n = 6) are indicated in red. USA300 isolates from a universal sample collection in Cambridgeshire (n = 24) are indicated in orange [24] and from the US (n = 348) in blue [25].

Mid-point rooted maximum likelihood tree based on SNPs in the core genome alignment generated after mapping against the reference genome FPR3757 (black).

**Figure 6:**
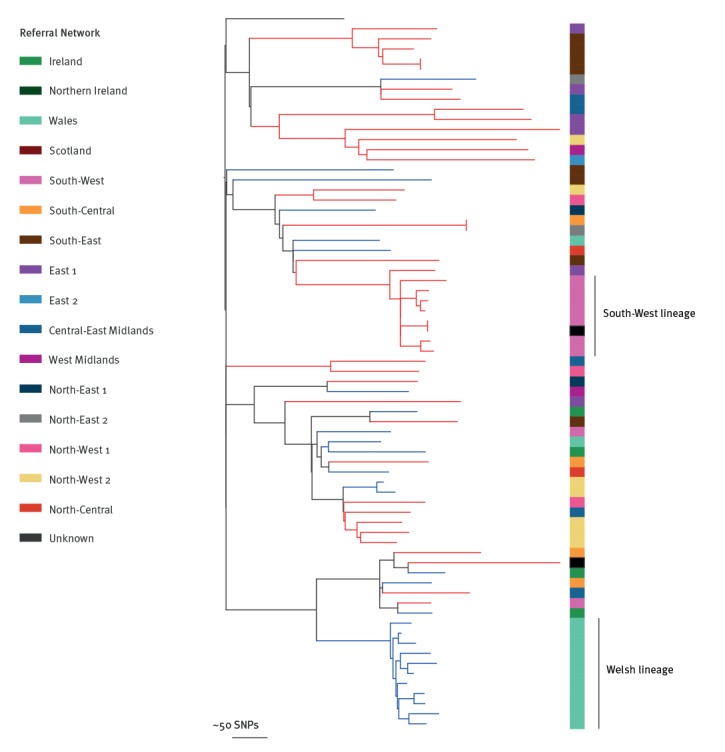
Phylogenetic tree showing PHE CC5 bloodstream infection isolates, 1 October 2012–30 September 2013 (n=42), and CC5 isolates from the previously published BSAC collection of bloodstream infections, 2000–2010 (n=28)

BSAC: British Society of Antimicrobial Chemotherapy; BSI: blood stream infections; CC: clonal complex; PHE: Public Health England; SNPs: single nucleotide polymorphisms.

Another potential benefit of having access to national surveillance data is the ability to identify and explore changes in molecular epidemiology on a local scale. By way of an example, we found an expansion of CC5 in South West England (Figure 6), which was genetically distinct from a CC5 expansion in Wales identified in the BSAC collection [13], despite their close geographic proximity.

PHE BSI CC5 isolates (n = 42) indicated by red branches. National BSI BSAC collection (n = 28) indicated by blue branches [13].

Mid-point rooted maximum likelihood tree based on SNPs in the core genome alignment generated after mapping against the CC5 reference genome N315. Coloured bar represents referral network of submitting laboratory. Expansions within the South West England and Welsh regions are highlighted.

### Backward compatibility of typing methods

Globally, PCR determination of *spa*-type is a commonly used typing method. However, as some laboratories transition to WGS-based typing, it is important that typing methods remain compatible. We examined the concordance between these two methods in the national MRSA BSI collection. Of the 425 isolates we found a 98.4% concordance rate (Supplementary Table S2), comparable to previous studies [33,34]. Of the seven isolates with discordant results, there were deletions/rearrangements within the *spa* gene of the short-read assemblies that resulted in loss of sequence complementary to forward primer, and thus failure to *in silico* amplify the gene region targeted by genomic *spa*-typing.

## Discussion

Mandatory enhanced surveillance for MRSA BSI in England has provided in-depth information on the national decline of MRSA BSI and the changes in patient-level epidemiology that have accompanied it [2]. However, without characterisation of systematically collected isolates, bacterial molecular epidemiology cannot be studied. This study aimed to investigate whether it was feasible to undertake combined epidemiological and genomic surveillance of MRSA bloodstream infections in England in order to address this issue.

We encountered some challenges including obtaining bloodstream isolates from all participating hospitals (as submission was voluntary) and integrating two datasets collected through different methods (epidemiological data collected through an online database submission and isolates sent with written information via post/courier). Despite this, we demonstrated the feasibility of this approach. We were able to construct the known population structure and diversity of MRSA in England, even with an incomplete collection of bloodstream isolates collected over a 1-year period. We found a greater diversity of clones than that seen in a 10-year national collection of MRSA bloodstream isolates (BSAC collection) with a limited sampling strategy, but less diversity than that seen in a 1-year regional collection of carriage and clinical isolates (Cambridgeshire study [24]). A sensible first step in MRSA surveillance is to assess existing genomic diversity [16] and our study demonstrates that this can be achieved and could feasibly be extended over time to generate a comprehensive national genomic database to monitor changes in clonal diversity.

Prior to April 2017, all MRSA BSI isolates submitted to PHE were routinely characterised by *spa*-typing and PCR to confirm species identification alongside determination of *mecA/C* and *luk-PV* status [17,18]. As typing methods evolve and WGS becomes increasingly routine, backward compatibility with previous methods ensures the continued utility of typed historical collections. Laboratory *spa*-typing and *spa*-genotyping from short read WGS data have been shown to be largely comparable in a limited number of studies [33,34], despite the high density of repeats within the *spa* gene region. We showed over 98% concordance between laboratory and genomic *spa*-typing methods which, reassuringly, confirms compatibility with historical data.

A further potential benefit of prospective sequencing of MRSA bloodstream isolates and a centralised national database is the ability to provide genomic context to confirm or refute outbreaks on a local or a national scale. This would be an invaluable resource as long as there is open access to anonymised (non-identifiable) data and to bioinformatics tools to analyse them rapidly and easily. The challenges will be to ensure standardised methods, development of strategies to avoid duplication of samples and establishment of a large, comprehensive, open access, anonymised database where data could be deposited, curated and accessible for public health benefit. Web-based, open-access software packages that are potentially suitable for this purpose are already being developed [35,36]. Apart from the ability to detect emerging or potentially high-risk MRSA clones retrospectively, on-going sampling and analysis will enable detection in real-time.

In this study, we found that that the high-risk USA300 lineage, an epidemic cause of SSTI in the US [26], has spread to the UK and is causing bloodstream infections across England. While the genomic data suggest multiple introductions of USA300, the use of BSI rather than clinical isolate-based surveillance limits our ability to analyse this further. However, using the PHE BSI collection it was possible to identify a local expansion of CC5 causing BSI in South West England, where local investigations suggest this clone has been causing excess disease [37]. Thus, timely, routine WGS of PHE BSI isolates combined with local epidemiological data could potentially identify novel and/or pathogenic lineages in real time and could be used to trigger local /regional investigations and interventions.

A major advantage of sequencing MRSA isolates is the ability to share and collate genome sequence data to build up national and international databases. A number of BSI surveillance systems already exist e.g. the English mandatory enhanced surveillance system, the voluntary British Society of Antimicrobial Chemotherapy BSI Surveillance Programme and the voluntary European Antimicrobial Resistance Surveillance Network. While each system has different aims and objectives, sampling criteria and data collection methods, the digital interchangeability of sequence data creates an opportunity to collaborate and share genome sequence data while producing a sustainable, on-going resource if the isolates were sequenced. The challenges will be to ensure standardised methods, development of strategies to avoid duplication of samples and establishment of a large, comprehensive, open-access database where anonymised data could be deposited, curated, and accessible for public health benefit.

We acknowledge several limitations in our study. The systems for collecting epidemiological data and bacterial isolates were separate and different, leading to high rates of sample exclusion. This challenge of capturing and integrating both types of data could be overcome in practice by submitting epidemiological and laboratory data to a single data collection system. Submission of bloodstream isolates was voluntary, with many reported cases having no corresponding isolate referred for characterisation; this may have introduced bias into the analysis. This could be addressed by having mandatory submission of isolates for all reported cases. Finally, we did not conduct a cost/benefit analysis of this approach. Despite these limitations, we have demonstrated that prospective epidemiological and genomic surveillance of MRSA bloodstream infections is feasible, has numerous potential benefits and could provide a valuable public health resource in England and beyond.

## Supplementary Data

260000 Supplementary Figure S1

## Supplementary Data

773000 Supplementary Table S1

## Supplementary Data

118000 Supplementary Table S2
