# Supplementary material for: Prospective genomic surveillance of methicillin-resistant Staphylococcus aureus (MRSA) associated with bloodstream infection, England, 1 October 2012 to 30 September 2013
Source: Euro Surveill. 2019 Jan 24;24(4):1800215. doi: 10.2807/1560-7917.ES.2019.24.4.1800215 (PMC6351993; doi:10.2807/1560-7917.ES.2019.24.4.1800215)

This supplementary material is hosted by *Eurosurveillance* as supporting information alongside the article 'Prospective genomic surveillance of methicillin-resistant Staphylococcus aureus (MRSA) associated with bloodstream infection, England, 1 October 2012 to 30 September 2013' on behalf of the authors who remain responsible for the accuracy and appropriateness of the content. The same standards for ethics, copyright, attributions and permissions as for the article apply. *Eurosurveillance* is not responsible for the maintenance of any links or email addresses provided therein.

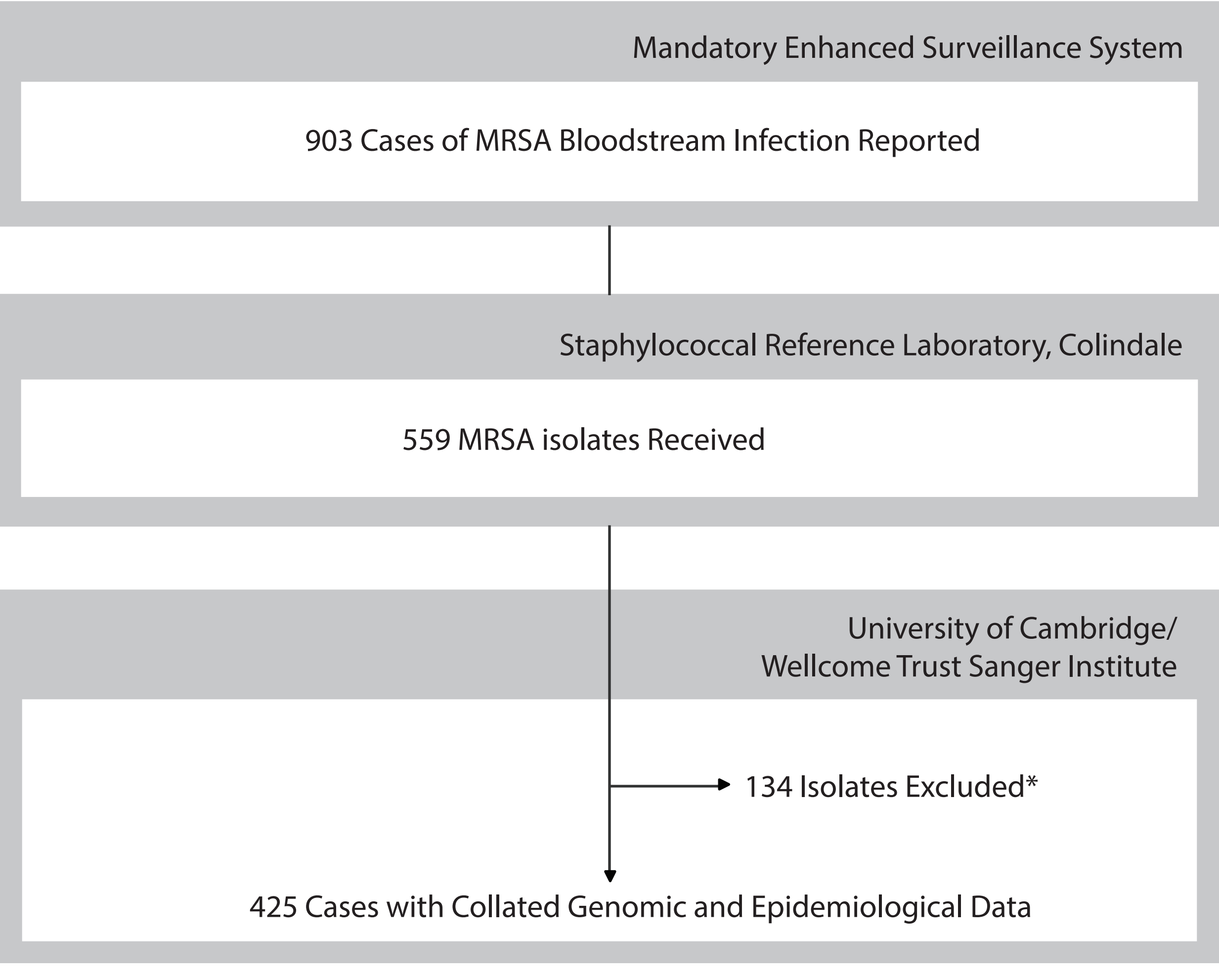

Supplement: Supplementary Figure S1 [file 1800215_TOLEMAN_SupplementaryFigureS1.pdf]
