# Supplementary material for: Prospective genomic surveillance of methicillin-resistant Staphylococcus aureus (MRSA) associated with bloodstream infection, England, 1 October 2012 to 30 September 2013
Source: Euro Surveill. 2019 Jan 24;24(4):1800215. doi: 10.2807/1560-7917.ES.2019.24.4.1800215 (PMC6351993; doi:10.2807/1560-7917.ES.2019.24.4.1800215)
Supplement: Supplementary Table S1 [file 1800215_TOLEMAN_SupplementaryTableS1.pdf]

Supplementary Table S1: Accession numbers and assembly statistics for isolates.

This supplementary material is hosted by Eurosurveillance as supporting information alongside the article 'Prospective genomic surveillance of methicillin-resistant Staphylococcus aureus (MRSA) associated with bloodstream infection, England, 1 October 2012 to 30 September 2013' on behalf of the authors who remain responsible for the accuracy and appropriateness of the content. The same standards for ethics, copyright, attributions and permissions as for the article apply. Eurosurveillance is not responsible for the maintenance of any links or email addresses provided therein

Lane	Accession Number	Assembly Type	Total Length	No Contigs	Avg Contig Length	Largest Contig	N50	Contigs in N50	N60	Contigs in N60	N70	Contigs in N70	N80	Contigs in N80	N90	Contigs in N90	N100	Contigs in N100	No scaffolded bases (N)
12483_8_74	ERR527291	Scaffold: Velvet + Improvement	2805786	29	96751.24	578294	406874	3	170600	5	125440	7	111803	9	54546	13	622	29	1196
12483_8_17	ERR527235	Scaffold: Velvet + Improvement	2785153	23	121093.61	710580	353665	3	294725	4	255470	5	98378	7	53903	11	424	23	3037
12625_1_22	ERR541004	Scaffold: Velvet + Improvement	2789012	21	132810.1	680362	351019	3	217869	4	191247	5	120286	7	73400	10	619	21	3638
12483_8_71	ERR527288	Scaffold: Velvet + Improvement	2827284	35	80779.54	336875	158444	7	128316	9	112928	11	88611	14	45010	18	402	35	562
12625_1_23	ERR541005	Scaffold: Velvet + Improvement	2786258	25	111450.32	410246	215664	5	210911	6	146335	8	125431	10	94511	12	307	25	940
12625_1_20	ERR541002	Scaffold: Velvet + Improvement	2892874	39	74176.26	310563	156340	7	146420	9	125428	11	75455	13	43904	18	369	39	714
12625_1_17	ERR540999	Scaffold: Velvet + Improvement	2898342	35	82809.77	331780	174046	7	156382	8	117708	11	108248	13	44092	18	440	35	789
12483_8_73	ERR527290	Scaffold: Velvet + Improvement	2783743	27	103101.59	430856	243617	5	224281	6	159915	7	111366	10	72633	13	472	27	2577
12483_8_69	ERR527286	Scaffold: Velvet + Improvement	2904264	22	132012	712694	239538	4	212021	5	173302	7	145552	8	106766	11	318	22	3187
12483_8_61	ERR527278	Scaffold: Velvet + Improvement	2800478	33	84862.97	406382	162908	6	133567	8	119356	10	106816	12	58841	16	385	33	1207
12625_1_24	ERR541006	Scaffold: Velvet + Improvement	2791957	39	71588.64	328884	129459	7	114536	9	108122	12	93835	15	49876	19	449	39	1041
12625_1_25	ERR541007	Scaffold: Velvet + Improvement	2954957	44	67158.11	355694	150949	7	128734	9	84359	12	64166	16	39949	22	400	44	3043
12625_1_13	ERR540995	Scaffold: Velvet + Improvement	2748820	26	105723.85	578300	215897	4	197064	5	146498	7	125434	9	74714	11	307	26	1483
12483_8_45	ERR527262	Scaffold: Velvet + Improvement	2814642	23	122375.74	442336	233965	4	176882	6	156388	8	135382	9	89374	12	411	23	826
12483_8_31	ERR527249	Scaffold: Velvet + Improvement	2849775	35	81422.14	334674	150786	6	143213	8	124223	11	102150	13	79966	16	307	35	1962
12483_8_68	ERR527285	Scaffold: Velvet + Improvement	2808044	40	70201.1	307929	155404	7	125447	9	104812	11	81398	14	40784	20	307	40	1616
12483_8_47	ERR527264	Scaffold: Velvet + Improvement	2951415	52	56757.98	252105	150360	8	99642	11	78492	14	60175	19	39065	25	309	52	7668
12483_8_21	ERR527239	Scaffold: Velvet + Improvement	2782501	26	107019.27	526565	176875	5	170549	7	131023	9	111723	11	55288	14	429	26	253
12483_8_30	ERR527248	Scaffold: Velvet + Improvement	2827457	29	97498.52	654368	258448	4	173962	5	156335	6	108246	9	56802	12	307	29	1181
12483_8_78	ERR527295	Scaffold: Velvet + Improvement	2825890	28	100924.64	340344	176999	6	174022	7	133555	9	118143	11	54689	15	385	28	1696
12483_8_20	ERR527238	Scaffold: Velvet + Improvement	2827598	29	97503.38	499812	167026	6	125412	8	118141	10	93937	13	54696	17	383	29	1590
12483_8_19	ERR527237	Scaffold: Velvet + Improvement	2743133	25	109725.32	493748	156373	5	146334	7	123021	9	108834	11	69239	14	419	25	261
12673_8_85	ERR555084	Scaffold: Velvet + Improvement	2822513	26	108558.19	360569	245050	5	237462	6	158036	8	125429	10	109187	12	406	26	308
12625_1_26	ERR541008	Scaffold: Velvet + Improvement	2946331	41	71861.73	354825	199458	6	150853	8	85094	10	62643	14	39944	20	327	41	2360
12625_1_18	ERR541000	Scaffold: Velvet + Improvement	2805752	24	116906.33	536809	215896	4	211414	5	185710	6	125429	8	77907	11	385	24	1332
12625_1_29	ERR541011	Scaffold: Velvet + Improvement	2810808	29	96924.41	540606	167877	5	146610	6	108262	9	99408	11	58163	15	415	29	1377
12483_8_77	ERR527294	Scaffold: Velvet + Improvement	2831213	28	101114.75	406522	211865	5	173860	7	146517	9	125526	11	64716	13	442	28	1950
12625_1_11	ERR540993	Scaffold: Velvet + Improvement	2777822	24	115742.58	517112	304025	4	227796	5	168990	7	94102	9	44032	13	378	24	1585
12625_1_8	ERR540990	Scaffold: Velvet + Improvement	2806852	24	116952.17	857011	236909	3	183501	5	174143	6	146401	8	108210	10	507	24	2250
12625_1_19	ERR541001	Scaffold: Velvet + Improvement	2827698	29	97506.83	426625	216095	5	185718	6	146380	8	125429	10	50736	14	652	29	3636
12483_8_49	ERR527266	Scaffold: Velvet + Improvement	2845038	28	101608.5	637106	339932	3	156368	4	131577	6	89581	9	45888	13	414	28	1255
12483_8_58	ERR527275	Scaffold: Velvet + Improvement	2795526	42	66560.14	363382	170598	6	144099	8	125438	10	82958	12	40103	17	373	42	1429
12483_8_35	ERR527253	Scaffold: Velvet + Improvement	2841639	59	48163.37	395482	133886	7	109648	9	69023	13	54712	17	37975	24	318	59	487
12483_8_75	ERR527292	Scaffold: Velvet + Improvement	2826913	26	108727.42	449011	213417	5	187355	6	156242	8	125425	10	94836	12	393	26	1237
12673_8_86	ERR555085	Scaffold: Velvet + Improvement	2834147	39	72670.44	548373	226039	4	163742	6	131944	8	102637	10	36932	15	572	39	1788
12483_8_25	ERR527243	Scaffold: Velvet + Improvement	2813209	31	90748.68	406445	222865	5	173975	6	146481	8	125428	10	57957	13	315	31	1795
12483_8_24	ERR527242	Scaffold: Velvet + Improvement	2840856	35	81167.31	289623	173973	6	160262	8	121638	10	91488	13	55057	17	307	35	1079
12625_1_9	ERR540991	Scaffold: Velvet + Improvement	2808869	25	112354.76	597681	364517	3	252234	4	170548	6	114148	8	88771	10	553	25	2127
12625_1_10	ERR540992	Scaffold: Velvet + Improvement	2811947	8	351493.38	1296603	1041310	2	1041310	2	1041310	2	1041310	2	116658	4	432	8	1669
12673_8_87	ERR555086	Scaffold: Velvet + Improvement	2915873	31	94060.42	410368	247491	5	153861	6	119167	8	108070	11	85829	14	373	31	2642
12483_8_65	ERR527282	Scaffold: Velvet + Improvement	2799433	33	84831.3	439284	149389	5	127839	7	108823	9	73672	13	39181	17	379	33	555
12625_1_5	ERR540987	Scaffold: Velvet + Improvement	2796225	31	90200.81	339248	167285	6	156345	8	132763	10	125439	12	54722	15	307	31	1517
12483_8_76	ERR527293	Scaffold: Velvet + Improvement	2830889	39	72586.9	340455	176721	6	173971	7	125431	9	85003	12	46064	16	302	39	1023
12483_8_87	ERR527304	Scaffold: Velvet + Improvement	2891127	44	65707.43	310892	146370	7	125423	9	119348	11	62849	16	41118	21	358	44	898
12483_8_88	ERR527305	Scaffold: Velvet + Improvement	2792389	18	155132.72	1164948	381609	2	376248	3	244013	4	242092	5	122862	6	855	18	1754
12483_8_93	ERR527310	Scaffold: Velvet + Improvement	2914920	30	97164	449946	343074	4	288902	5	247375	6	109345	8	54184	12	350	30	2263
12483_8_2	ERR527220	Scaffold: Velvet + Improvement	2757398	25	110295.92	490637	253102	4	146419	6	125473	8	108246	10	57964	14	1992	25	280
12483_8_52	ERR527269	Scaffold: Velvet + Improvement	2885056	36	80140.44	334661	174026	6	146418	8	115705	10	108239	13	58137	16	307	36	1598
12483_8_83	ERR527300	Scaffold: Velvet + Improvement	2865262	42	68220.52	448536	211065	5	156377	6	131424	8	73524	11	39376	16	359	42	1385
12673_8_88	ERR555087	Scaffold: Velvet + Improvement	2790152	29	96212.14	1043673	527546	2	234051	3	232556	4	220055	5	106059	8	368	29	954
12483_8_28	ERR527246	Scaffold: Velvet + Improvement	2854727	28	101954.54	406361	178507	6	173982	7	146642	9	125438	12	55086	15	406	28	545
12625_1_14	ERR540996	Scaffold: Velvet + Improvement	2832768	32	88524	348864	173980	6	156375	7	130789	9	86923	12	52178	16	385	32	1759
12625_1_3	ERR540985	Scaffold: Velvet + Improvement	2825318	14	201808.43	548283	481656	3	463965	4	463965	4	331578	5	108470	7	627	14	1515
12483_8_16	ERR527234	Scaffold: Velvet + Improvement	2799500	27	103685.19	538456	170552	6	158715	7	108840	10	96550	12	65165	16	393	27	1472
12483_8_55	ERR527272	Scaffold: Velvet + Improvement	2803664	33	84959.52	404559	176944	5	174569	6	155241	8	108596	10	47818	14	386	33	954
12483_8_81	ERR527298	Scaffold: Velvet + Improvement	2936789	45	65261.98	253394	130847	8	83656	11	77910	15	62665	19	39061	25	421	45	1798
12625_1_27	ERR541009	Scaffold: Velvet + Improvement	2833560	25	113342.4	572935	183521	5	162576	7	146325	9	125435	11	88798	13	307	25	1011
12483_8_51	ERR527268	Scaffold: Velvet + Improvement	2861902	45	63597.82	371523	170377	6	137328	8	128612	11	104120	13	56341	17	395	45	3489
12483_8_4	ERR527222	Scaffold: Velvet + Improvement	2798288	30	93276.27	312701	146422	7	137034	9	123663	11	103693	14	54477	17	307	30	1764
12483_8_56	ERR527273	Scaffold: Velvet + Improvement	2866158	35	81890.23	334632	156330	6	125449	8	113225	11	68796	14	57727	18	410	35	1185
12483_8_32	ERR527250	Scaffold: Velvet + Improvement	2827021	27	104704.48	532247	252836	4	187257	6	176868	7	125477	10	55100	12	319	27	867
12483_8_92	ERR527309	Scaffold: Velvet + Improvement	2826453	30	94215.1	335183	156386	6	146276	8	130647	10	108241	13	57719	16	307	30	1075

12483_8_54	ERR527271	Scaffold: Velvet + Improvement	2787691	28	99560.39	400339	254900	5	146344	6	124151	9	108245	11	42135	15	653	28	1458
12483_8_95	ERR527312	Scaffold: Velvet + Improvement	2857819	33	86600.58	318899	173966	6	155502	8	130624	10	125275	12	46031	16	393	33	1736
12483_8_59	ERR527276	Scaffold: Velvet + Improvement	2844079	31	91744.48	406643	187194	5	174238	7	146279	9	105474	11	50638	15	381	31	574
12483_8_1	ERR527219	Scaffold: Velvet + Improvement	2800708	34	82373.76	530265	173960	5	170781	6	141655	8	111757	10	41834	14	307	34	817
12483_8_11	ERR527229	Scaffold: Velvet + Improvement	2823612	48	58825.25	404637	148370	6	125406	8	108129	10	63454	14	40099	19	438	48	610
12625_1_2	ERR540984	Scaffold: Velvet + Improvement	2857227	39	73262.23	312292	150786	7	146400	8	108286	11	86923	14	53447	18	307	39	769
12625_1_15	ERR540997	Scaffold: Velvet + Improvement	2805203	26	107892.42	531589	174151	4	154883	6	146397	7	95857	10	81340	13	307	26	1491
12483_8_41	ERR527258	Scaffold: Velvet + Improvement	2802200	27	103785.19	539518	246114	4	173964	6	170546	7	125428	9	73324	12	442	27	2266
12483_8_60	ERR527277	Scaffold: Velvet + Improvement	2794613	27	103504.19	579949	386206	3	177328	5	167348	6	129826	8	56841	12	526	27	2252
12483_8_62	ERR527279	Scaffold: Velvet + Improvement	2800725	27	103730.56	406345	173968	5	162958	7	132032	9	111708	11	73434	14	307	27	1092
12625_1_7	ERR540989	Scaffold: Velvet + Improvement	2870348	29	98977.52	335085	174567	6	173362	7	125447	9	94837	12	54697	16	656	29	1299
12483_8_15	ERR527233	Scaffold: Velvet + Improvement	2808856	14	200632.57	1240701	544212	2	544212	2	243983	3	221220	4	124229	6	472	14	1751
12483_8_7	ERR527225	Scaffold: Velvet + Improvement	2761492	29	95223.86	333533	176831	6	158677	8	125431	10	110153	12	57671	15	653	29	1919
12625_1_4	ERR540986	Scaffold: Velvet + Improvement	2757384	27	102125.33	585437	407252	3	170557	5	125437	7	108272	9	63012	12	307	27	671
12483_8_80	ERR527297	Scaffold: Velvet + Improvement	2780864	16	173804	1012881	318029	3	295843	4	295843	4	144663	6	93848	8	385	16	1584
12483_8_66	ERR527283	Scaffold: Velvet + Improvement	2782358	24	115931.58	625639	224810	4	174874	6	145795	8	135529	10	80139	12	450	24	1671
12483_8_90	ERR527307	Scaffold: Velvet + Improvement	2731764	17	160692	826301	771413	2	378870	3	378870	3	325567	4	81952	6	366	17	2309
12483_8_53	ERR527270	Scaffold: Velvet + Improvement	2829556	30	94318.53	417747	190560	5	170629	7	125436	9	108197	11	54689	15	443	30	1858
12483_8_34	ERR527252	Scaffold: Velvet + Improvement	2823203	33	85551.61	410658	173968	5	146506	7	119389	9	98654	12	45434	16	376	33	1314
12625_1_1	ERR540983	Scaffold: Velvet + Improvement	2831886	31	91351.16	406961	212595	5	156326	7	134066	9	108250	11	50708	15	449	31	1193
12483_8_86	ERR527303	Scaffold: Velvet + Improvement	2945102	48	61356.29	355132	170904	6	150359	8	83264	11	73294	15	39092	20	345	48	3820
12483_8_79	ERR527296	Scaffold: Velvet + Improvement	2746042	24	114418.42	662946	283591	4	169860	5	129369	7	90401	9	44033	13	709	24	2570
12483_8_9	ERR527227	Scaffold: Velvet + Improvement	2841545	30	94718.17	432115	219258	5	170536	7	132631	9	125418	11	54970	14	435	30	839
12483_8_14	ERR527232	Scaffold: Velvet + Improvement	2784489	33	84378.45	360278	169827	5	135666	7	85889	10	77638	13	38037	18	865	33	1219
12483_8_63	ERR527280	Scaffold: Velvet + Improvement	2745413	16	171588.31	957389	324483	3	322874	4	322874	4	291652	5	269767	6	413	16	1203
12483_8_29	ERR527247	Scaffold: Velvet + Improvement	2904003	47	61787.3	253154	145784	8	124513	11	85192	13	60196	18	34385	24	318	47	3498
12483_8_82	ERR527299	Scaffold: Velvet + Improvement	2859712	40	71492.8	406750	172821	6	125435	8	97545	11	67235	14	49143	19	395	40	861
12483_8_3	ERR527221	Scaffold: Velvet + Improvement	2852804	31	92025.94	339070	172516	6	150426	8	106796	10	69300	14	54599	18	409	31	4013
12483_8_57	ERR527274	Scaffold: Velvet + Improvement	2784827	27	103141.74	445853	211703	5	173966	7	156334	8	131551	10	108263	12	307	27	757
12483_8_38	ERR527256	Scaffold: Velvet + Improvement	2787729	24	116155.38	443567	236587	4	180658	6	176886	7	125428	9	108243	12	445	24	1421
12483_8_42	ERR527259	Scaffold: Velvet + Improvement	2769530	33	83925.15	528506	166712	6	129463	8	109149	10	97873	13	54657	17	473	33	809
12483_8_36	ERR527254	Scaffold: Velvet + Improvement	2726814	25	109072.56	333191	176658	6	167230	8	164173	9	117611	11	65978	14	657	25	847
12483_8_67	ERR527284	Scaffold: Velvet + Improvement	2782288	25	111291.52	406563	213079	5	211790	6	173967	8	125434	10	57673	13	307	25	1132
12625_1_28	ERR541010	Scaffold: Velvet + Improvement	2874153	33	87095.55	443505	216245	5	174006	6	130872	8	116620	10	48124	14	388	33	1498
12483_8_50	ERR527267	Scaffold: Velvet + Improvement	2803347	30	93444.9	406537	173991	5	146171	6	123641	8	94841	11	56774	15	442	30	665
12483_8_89	ERR527306	Scaffold: Velvet + Improvement	2741502	15	182766.8	607240	440589	3	440589	3	325109	4	156789	6	110568	8	464	15	1701
12625_1_6	ERR540988	Scaffold: Velvet + Improvement	2795756	30	93191.87	625574	174882	5	138476	7	109861	9	90579	12	61421	16	541	30	2168
12755_8_68	ERR564224	Scaffold: Velvet + Improvement	2821412	26	108515.85	511707	244583	5	222088	6	172338	7	133634	9	83621	12	401	26	1275
12483_8_94	ERR527311	Scaffold: Velvet + Improvement	2884841	41	70361.98	334668	178065	6	125416	9	115690	11	71148	14	41117	19	339	41	1171
12483_8_84	ERR527301	Scaffold: Velvet + Improvement	2803537	36	77876.03	348764	165123	6	125447	8	96459	11	74745	14	44625	19	440	36	698
12483_8_26	ERR527244	Scaffold: Velvet + Improvement	2768320	22	125832.73	1171791	349496	2	281913	3	269768	4	113798	6	81664	8	310	22	2642
12625_1_30	ERR541012	Scaffold: Velvet + Improvement	2952602	41	72014.68	354364	170808	7	150364	9	99442	11	67656	14	39066	20	430	41	3478
12625_1_32	ERR541014	Scaffold: Velvet + Improvement	2766459	16	172903.69	969638	766243	2	766243	2	243625	3	169652	5	136254	6	371	16	1896
12589_1_6	ERR540707	Scaffold: Velvet + Improvement	2828482	24	117853.42	449190	211516	5	183165	7	174162	8	146339	10	108259	12	649	24	806
12625_1_33	ERR541015	Scaffold: Velvet + Improvement	2853499	33	86469.67	581872	210562	5	146504	6	125433	8	89207	11	41751	16	366	33	1110
12625_1_34	ERR541016	Scaffold: Velvet + Improvement	2847832	19	149885.89	665585	222391	4	219041	5	203493	6	122263	8	84296	11	371	19	3348
12625_1_35	ERR541017	Scaffold: Velvet + Improvement	2789449	39	71524.33	252887	122483	9	108282	11	93838	14	59705	18	39381	23	344	39	1548
12625_1_36	ERR541018	Scaffold: Velvet + Improvement	2789751	28	99633.96	520827	176076	5	169868	6	146344	8	112375	10	43430	14	529	28	1096
12625_1_37	ERR541019	Scaffold: Velvet + Improvement	2764775	27	102399.07	403134	187674	5	174254	7	156326	8	110265	11	75007	13	307	27	2039
12625_1_38	ERR541020	Scaffold: Velvet + Improvement	2795876	15	186391.73	1259426	868969	2	868969	2	170584	3	92445	6	340	15	3795		
12625_1_42	ERR541023	Scaffold: Velvet + Improvement	2816327	24	117346.96	641784	253084	4	176881	6	173971	7	125434	9	94844	11	651	24	1367
12625_1_43	ERR541024	Scaffold: Velvet + Improvement	2817700	11	256154.55	1396697	607955	2	607955	2	607955	2	329700	3	127662	5	316	11	2219
12625_1_45	ERR541026	Scaffold: Velvet + Improvement	2815609	30	93853.63	561845	236672	4	177640	6	173981	7	125429	9	42658	13	401	30	1030
12625_1_46	ERR541027	Scaffold: Velvet + Improvement	2826754	29	97474.28	406603	215892	5	146603	7	128341	9	108724	11	68529	14	385	29	1645
12625_1_47	ERR541028	Scaffold: Velvet + Improvement	2757611	26	106061.96	502337	176883	4	173972	5	125443	7	108255	9	76346	12	446	26	1488
12625_1_48	ERR541029	Scaffold: Velvet + Improvement	2880371	31	92915.19	334637	176580	6	174006	7	139775	9	125442	11	42557	16	473	31	1134
12625_1_49	ERR541030	Scaffold: Velvet + Improvement	2817986	38	74157.53	312907	169524	7	156434	9	113815	11	92660	14	54552	18	307	38	2295
12625_1_53	ERR541034	Scaffold: Velvet + Improvement	2788288	27	103269.93	539951	406586	3	174627	5	125432	7	108880	9	80113	12	307	27	706
12625_1_54	ERR541035	Scaffold: Velvet + Improvement	2818505	24	117437.71	406733	168653	6	142682	8	125435	10	88329	12	57962	16	971	24	1957
12625_1_50	ERR541031	Scaffold: Velvet + Improvement	2841089	33	86093.61	335264	190443	6	156378	7	125489	9	111240	12	66790	15	307	33	611
12625_1_51	ERR541032	Scaffold: Velvet + Improvement	2837131	23	123353.52	406750	211884	5	173974	7	170567	8	125433	10	69185	13	584	23	2003
12625_1_52	ERR541033	Scaffold: Velvet + Improvement	2869983	40	71749.57	334427	174163	6	146511	8	125427	10	88665	13	41237	18	327	40	834
12625_1_55	ERR541036	Scaffold: Velvet + Improvement	2876246	19	151381.37	609282	421538	3	346428	4	326134	5	175070	6	109342	8	1105	19	3217
12625_1_56	ERR541037	Scaffold: Velvet + Improvement	2833519	33	85864.21	299986	159979	7	131412	9	112277	11	80406	14	44746	19	451	33	2028
12625_1_57	ERR541038	Scaffold: Velvet + Improvement	2847960	26	109536.92	433732	336060	4	211548	5	176589	7	116201	9	66997	12	302	26	1331
12625_1_59	ERR541040	Scaffold: Velvet + Improvement	2833232	28	101186.86	325018	190898	6	176820	7	133971	9	87749	12	52181	16	448	28	605
12625_1_60	ERR541041	Scaffold: Velvet + Improvement	2799996	22	127272.55	485656	236281	4	219193	5	178933	7	156335	9	108277	11	653	22	1695

12625_1_61	ERR541042	Scaffold: Velvet + Improvement	2784951	27	103146.33	401456	211829	5	190852	6	156330	8	146499	10	94927	12	307	27	1710
12625_1_62	ERR541043	Scaffold: Velvet + Improvement	2758550	21	131359.52	601319	254816	3	174250	5	170595	6	139590	8	109042	10	445	21	1454
12625_1_63	ERR541044	Scaffold: Velvet + Improvement	2838019	26	109154.58	623662	190859	4	173976	6	156338	7	125441	9	66401	12	362	26	985
12625_1_64	ERR541045	Scaffold: Velvet + Improvement	2802106	29	96624.34	296088	183393	6	174027	8	132427	10	108875	12	58684	15	329	29	1615
12625_1_65	ERR541046	Scaffold: Velvet + Improvement	2768537	15	184569.13	1004355	574665	2	381735	3	381735	3	346730	4	74798	6	516	15	1894
12589_1_7	ERR540708	Scaffold: Velvet + Improvement	2863180	47	60918.72	326481	174569	6	146334	8	125436	10	108247	12	41800	17	307	47	991
12625_1_66	ERR541047	Scaffold: Velvet + Improvement	2829811	23	123035.26	521506	211546	5	173864	6	125440	8	79567	11	59109	15	653	23	1552
12625_1_67	ERR541048	Scaffold: Velvet + Improvement	2929073	42	69739.83	323473	150304	8	120163	10	79290	13	67660	17	54597	21	427	42	3050
12625_1_69	ERR541050	Scaffold: Velvet + Improvement	2889124	48	60190.08	307193	148843	7	148382	8	112236	11	74078	14	40098	19	326	48	1168
12625_1_70	ERR541051	Scaffold: Velvet + Improvement	2795704	21	133128.76	532752	229302	5	229157	6	190267	7	176996	9	121593	11	653	21	2227
12625_1_71	ERR541052	Scaffold: Velvet + Improvement	2812244	28	100437.29	495521	237888	4	211458	5	168463	7	135584	9	108257	11	307	28	2770
12625_1_72	ERR541053	Scaffold: Velvet + Improvement	2728718	15	181914.53	1290868	485615	2	485615	2	321306	3	227094	4	172928	5	342	15	2478
12625_1_73	ERR541054	Scaffold: Velvet + Improvement	2804579	28	100163.54	409160	183369	5	146434	7	125436	9	104043	12	54687	16	653	28	1386
12625_1_74	ERR541055	Scaffold: Velvet + Improvement	2821148	31	91004.77	534430	185704	5	168813	7	136133	9	114306	11	57958	15	307	31	1171
12625_1_75	ERR541056	Scaffold: Velvet + Improvement	2894237	19	152328.26	902815	616181	2	432692	3	347443	4	173502	5	106827	7	1651	19	1977
12625_1_76	ERR541057	Scaffold: Velvet + Improvement	2794704	24	116446	808154	398675	3	398675	3	172043	5	139310	6	104056	9	321	24	809
12625_1_78	ERR541059	Scaffold: Velvet + Improvement	2801895	14	200135.36	822244	644700	2	262458	3	246280	4	167887	6	108799	8	612	14	2572
12625_1_79	ERR541060	Scaffold: Velvet + Improvement	2892772	31	93315.23	480959	170894	6	150378	8	106834	10	70202	13	40132	18	421	31	1760
12625_1_80	ERR541061	Scaffold: Velvet + Improvement	2781800	18	154544.44	426918	305539	4	288903	5	270755	6	247897	7	135762	8	2484	18	1325
12625_1_81	ERR541062	Scaffold: Velvet + Improvement	2793711	14	199550.79	1043060	775249	2	775249	2	243955	3	243594	4	124326	6	1704	14	2519
12625_1_82	ERR541063	Scaffold: Velvet + Improvement	2932105	45	65157.89	354686	150369	8	120110	10	80093	13	63976	17	39067	23	361	45	2168
12625_1_83	ERR541064	Scaffold: Velvet + Improvement	2765934	38	72787.74	300044	156345	7	119681	9	110204	11	78079	14	46890	19	387	38	1253
12625_1_84	ERR541065	Scaffold: Velvet + Improvement	2808924	22	127678.36	426029	211888	5	173866	6	146458	8	117266	10	95660	12	647	22	758
12625_1_85	ERR541066	Scaffold: Velvet + Improvement	2809808	24	117075.33	406234	211846	5	177981	6	154204	8	125706	10	94840	13	307	24	927
12625_1_86	ERR541067	Scaffold: Velvet + Improvement	2774045	19	146002.37	622592	459067	3	426610	4	426610	4	325465	5	106670	7	396	19	787
12625_1_87	ERR541068	Scaffold: Velvet + Improvement	2792216	16	174513.5	1042516	398918	2	376154	3	243951	4	243556	5	122913	7	460	16	2368
12625_1_89	ERR541070	Scaffold: Velvet + Improvement	2886035	34	84883.38	335172	146433	7	116048	9	108620	12	69181	15	41417	20	449	34	1293
12625_1_90	ERR541071	Scaffold: Velvet + Improvement	2776797	48	57849.94	311566	156412	7	116370	9	106212	11	59426	15	39841	20	307	48	1580
12625_1_91	ERR541072	Scaffold: Velvet + Improvement	2830918	27	104848.81	447811	325298	4	187520	5	169158	7	125446	9	54693	12	406	27	1167
12625_1_92	ERR541073	Scaffold: Velvet + Improvement	2831198	28	101114.21	340338	174168	6	160814	8	128399	10	125314	12	57968	15	460	28	661
12625_1_93	ERR541074	Scaffold: Velvet + Improvement	2795747	30	93191.57	450586	179094	4	175357	6	146511	8	109208	10	54535	13	755	30	1796
12625_1_95	ERR541076	Scaffold: Velvet + Improvement	2870876	30	95695.87	601992	252299	4	170543	6	125416	8	110204	10	54695	14	449	30	2057
12593_1_1	ERR540795	Scaffold: Velvet + Improvement	2795381	17	164434.18	1028541	326924	3	326924	3	269859	4	169787	6	108541	8	413	17	2117
12593_1_2	ERR540796	Scaffold: Velvet + Improvement	2801344	33	84889.21	354667	170582	6	141375	8	131618	10	108773	12	52616	15	307	33	1243
12593_1_4	ERR540798	Scaffold: Velvet + Improvement	2818109	34	82885.56	406515	156349	6	146440	8	125442	10	77305	12	54673	17	377	34	1718
12593_1_64	ERR540857	Scaffold: Velvet + Improvement	2729381	15	181958.73	764747	727015	2	326035	3	214601	4	164464	5	81955	8	470	15	2317
12593_1_5	ERR540799	Scaffold: Velvet + Improvement	2802676	28	100095.57	406730	211567	5	170452	7	125431	9	110161	11	56837	14	307	28	1447
12593_1_6	ERR540800	Scaffold: Velvet + Improvement	2946472	54	54564.3	252177	150846	8	145771	10	96748	12	60178	16	39079	22	399	54	1697
12593_1_7	ERR540801	Scaffold: Velvet + Improvement	2843007	33	86151.73	334848	173924	7	170558	8	125437	10	96776	13	54680	17	402	33	561
12593_1_9	ERR540803	Scaffold: Velvet + Improvement	2802821	26	107800.81	407003	211279	5	176872	7	174156	8	125165	10	69016	13	649	26	734
12593_1_10	ERR540804	Scaffold: Velvet + Improvement	2817739	16	176108.69	1340092	326153	2	317480	3	317480	3	173679	5	78237	7	312	16	3150
12593_1_11	ERR540805	Scaffold: Velvet + Improvement	2775205	35	79291.57	406736	156331	6	146510	7	122463	10	87372	12	54693	16	408	35	1213
12593_1_13	ERR540807	Scaffold: Velvet + Improvement	2821445	29	97291.21	406652	311237	4	311229	5	126710	7	118652	9	50594	13	302	29	1821
12593_1_65	ERR540858	Scaffold: Velvet + Improvement	2993887	43	69625.28	322311	169300	7	113568	9	99432	12	80827	15	49290	20	442	43	1552
12593_1_15	ERR540809	Scaffold: Velvet + Improvement	2919161	21	139007.67	1229100	471733	2	346817	3	346817	3	153405	5	106048	8	548	21	2580
12593_1_16	ERR540810	Scaffold: Velvet + Improvement	2722167	16	170135.44	1058188	598629	2	598629	2	290071	3	163235	5	88293	7	371	16	1700
12593_1_17	ERR540811	Scaffold: Velvet + Improvement	2750792	35	78594.06	347780	224199	5	155112	6	134780	8	77695	11	34699	16	384	35	1619
12593_1_18	ERR540812	Scaffold: Velvet + Improvement	2835115	32	88597.34	406288	213528	5	146752	7	125430	9	88669	12	54566	16	653	32	8172
12593_1_19	ERR540813	Scaffold: Velvet + Improvement	2786940	33	84452.73	406171	160077	6	113626	8	109196	10	74489	14	54700	18	307	33	1000
12593_1_20	ERR540814	Scaffold: Velvet + Improvement	2812598	13	216353.69	1393898	326231	2	326231	2	316962	3	285519	4	127605	6	748	13	2214
12593_1_21	ERR540815	Scaffold: Velvet + Improvement	2791141	28	99683.61	422355	190485	5	159906	7	144081	9	110066	11	54977	14	442	28	757
12593_1_22	ERR540816	Scaffold: Velvet + Improvement	2854985	30	95166.17	406835	186271	5	174160	7	168591	8	125434	10	41135	15	557	30	997
12593_1_23	ERR540817	Scaffold: Velvet + Improvement	2883320	40	72083	310898	167257	7	131991	9	114266	11	67068	14	39385	20	307	40	1875
12593_1_24	ERR540818	Scaffold: Velvet + Improvement	2802547	32	87579.59	339999	190453	6	155462	8	125459	10	121371	12	43725	16	348	32	1413
12593_1_66	ERR540859	Scaffold: Velvet + Improvement	2927752	50	58555.04	326631	176815	6	163118	8	125448	10	108245	12	41236	17	315	50	996
12593_1_26	ERR540820	Scaffold: Velvet + Improvement	2875193	28	102685.46	335369	211429	6	173975	7	131964	9	115942	11	65808	14	390	28	3231
12593_1_27	ERR540821	Scaffold: Velvet + Improvement	2874023	35	82114.94	337092	173974	6	146989	8	125450	10	84349	12	41861	17	440	35	1729
12593_1_28	ERR540822	Scaffold: Velvet + Improvement	2881571	38	75830.82	338141	211446	5	170553	7	136166	9	113503	11	39726	16	305	38	2049
12589_1_8	ERR540709	Scaffold: Velvet + Improvement	2834628	27	104986.22	406502	215472	5	162926	7	131632	9	115444	11	55410	15	653	27	751
12593_1_29	ERR540823	Scaffold: Velvet + Improvement	2749113	10	274911.3	1291662	544089	2	544089	2	459667	3	459667	3	108395	5	3300	10	550
12593_1_30	ERR540824	Scaffold: Velvet + Improvement	2802886	38	73760.16	444231	211664	5	170549	7	131402	9	108247	11	54605	16	341	38	1164
12593_1_68	ERR540861	Scaffold: Velvet + Improvement	2743349	34	80686.74	406910	174028	6	156257	7	128565	9	72571	12	54694	16	401	34	1318
12593_1_69	ERR540862	Scaffold: Velvet + Improvement	2794465	28	99802.32	406743	211400	5	156339	7	131416	9	110182	11	54527	15	307	28	1393
12589_1_9	ERR540710	Scaffold: Velvet + Improvement	2875276	43	66866.88	739265	387692	3	245385	4	176048	5	137722	7	39017	11	407	43	2028
12589_1_10	ERR540711	Scaffold: Velvet + Improvement	2770288	17	162958.12	1047276	767560	2	767560	2	299563	3	88493	5	86395	8	384	17	2267
12589_1_11	ERR540712	Scaffold: Velvet + Improvement	2818356	14	201311.14	1418352	1418352	1	326191	2	318196	3	285085	4	108444	6	316	14	4505
12589_1_12	ERR540713	Scaffold: Velvet + Improvement	2861235	30	95374.5	495723	236603	4	173976	6	156333	7	125427	10	41385	13	440	30	1540

12593_1_31	ERR540825	Scaffold: Velvet + Improvement	2802526	18	155695.89	1024385	386162	2	363059	3	297059	4	190745	5	115320	7	425	18	2112
12589_1_13	ERR540714	Scaffold: Velvet + Improvement	2866277	36	79618.81	331774	156372	7	146347	9	125378	11	110001	14	44082	17	307	36	2114
12593_1_32	ERR540826	Scaffold: Velvet + Improvement	2822711	15	188180.73	1396871	594472	2	594472	2	594472	2	370115	3	107937	5	470	15	1948
12593_1_33	ERR540827	Scaffold: Velvet + Improvement	2829824	36	78606.22	390629	214664	5	173969	7	118564	9	100939	12	57477	15	305	36	2163
12589_1_17	ERR540718	Scaffold: Velvet + Improvement	2808564	33	85108	582327	187028	5	173972	7	156319	8	110206	10	54692	15	512	33	889
12593_1_72	ERR540865	Scaffold: Velvet + Improvement	2820532	28	100733.29	540157	264183	4	156234	6	125429	8	108863	10	54713	14	519	28	1062
12593_1_73	ERR540866	Scaffold: Velvet + Improvement	2817927	32	88060.22	406775	183667	5	156334	7	135893	9	125429	11	54832	15	388	32	2180
12589_1_19	ERR540720	Scaffold: Velvet + Improvement	2830610	31	91310	449024	187597	5	156368	7	131188	9	108341	11	75013	14	307	31	1900
12593_1_35	ERR540829	Scaffold: Velvet + Improvement	2852499	34	83897.03	354272	162431	6	146336	8	112474	10	107930	12	57903	16	404	34	987
12593_1_75	ERR540868	Scaffold: Velvet + Improvement	2758718	30	91957.27	314529	173974	6	133403	8	127270	10	95025	13	54552	17	307	30	760
12593_1_76	ERR540869	Scaffold: Velvet + Improvement	2845261	46	61853.5	356358	156399	7	122003	9	109659	11	88263	14	39289	20	305	46	3012
12589_1_20	ERR540721	Scaffold: Velvet + Improvement	2797283	12	233106.92	981412	611731	2	446123	3	446123	3	110563	5	102309	7	24368	12	803
12589_1_21	ERR540722	Scaffold: Velvet + Improvement	2798522	27	103648.96	414782	173970	5	169629	7	125419	9	108175	11	88626	14	310	27	1873
12589_1_22	ERR540723	Scaffold: Velvet + Improvement	2829945	31	91288.55	334701	176674	6	156328	7	125445	10	110202	12	57723	15	307	31	873
12589_1_50	ERR540751	Scaffold: Velvet + Improvement	2869407	50	57388.14	406663	174046	6	157139	8	108808	10	87821	13	39375	17	429	50	852
12593_1_37	ERR540831	Scaffold: Velvet + Improvement	2832872	31	91382.97	306085	269181	5	149272	7	90650	10	77658	13	38044	18	709	31	2034
12593_1_38	ERR540832	Scaffold: Velvet + Improvement	2803655	27	103839.07	406672	194148	5	176946	6	171441	8	125431	10	48150	13	303	27	2025
12589_1_23	ERR540724	Scaffold: Velvet + Improvement	2785469	28	99481.04	406826	173959	6	156334	7	133076	9	108884	11	65164	14	440	28	1587
12589_1_24	ERR540725	Scaffold: Velvet + Improvement	2791320	26	107358.46	383036	251837	5	233174	6	185109	7	117883	9	59872	12	609	26	1251
12593_1_77	ERR540870	Scaffold: Velvet + Improvement	2836038	31	91485.1	580810	257862	4	211455	5	125426	7	103780	10	54653	14	362	31	2531
12593_1_78	ERR540871	Scaffold: Velvet + Improvement	2906255	45	64583.44	275676	150854	7	95529	10	76228	13	63982	18	39069	23	421	45	1466
12593_1_79	ERR540872	Scaffold: Velvet + Improvement	2907021	45	64600.47	385288	150867	7	122488	9	85085	12	63976	16	42438	22	330	45	2282
12593_1_80	ERR540873	Scaffold: Velvet + Improvement	2799453	23	121715.35	428986	258032	4	170581	6	152775	7	131151	9	89854	12	307	23	2651
12589_1_51	ERR540752	Scaffold: Velvet + Improvement	2823033	31	91065.58	368196	174562	6	156381	7	124223	9	113644	12	46052	15	589	31	1688
12589_1_62	ERR540763	Scaffold: Velvet + Improvement	2858563	41	69721.05	272241	150354	7	140175	9	79667	12	62581	16	39967	21	413	41	2441
12755_8_70	ERR564226	Scaffold: Velvet + Improvement	2806489	35	80185.4	411683	173986	5	146351	7	125448	9	102223	12	42176	16	307	35	256
12593_1_40	ERR540833	Scaffold: Velvet + Improvement	2952223	30	98407.43	745217	256581	4	180868	5	141684	7	114515	9	54844	13	440	30	1163
12593_1_41	ERR540834	Scaffold: Velvet + Improvement	2773134	35	79232.4	349109	124766	7	108223	10	100454	12	73129	16	42886	21	613	35	1158
12593_1_42	ERR540835	Scaffold: Velvet + Improvement	2849098	55	51801.78	328287	156449	7	111797	10	97524	12	55112	16	39826	21	305	55	1843
12589_1_28	ERR540729	Scaffold: Velvet + Improvement	2734718	28	97668.5	525880	211643	4	158440	6	129469	8	108554	10	65372	13	358	28	785
12593_1_44	ERR540837	Scaffold: Velvet + Improvement	2808775	24	117032.29	409850	312987	4	236369	5	167982	7	132652	9	108285	11	307	24	2014
12593_1_45	ERR540838	Scaffold: Velvet + Improvement	2803545	31	90436.94	355325	187753	5	168886	7	134690	9	101019	11	73428	14	386	31	520
12593_1_46	ERR540839	Scaffold: Velvet + Improvement	2900348	41	70740.2	252177	150367	8	139190	10	124493	12	69985	15	39066	21	414	41	2977
12589_1_63	ERR540764	Scaffold: Velvet + Improvement	2744198	27	101636.96	356471	156386	6	136153	8	117596	10	90539	12	55130	16	447	27	728
12589_1_29	ERR540730	Scaffold: Velvet + Improvement	2791032	28	99679.71	581195	156333	5	153509	7	135629	9	124518	11	54697	14	307	28	670
12593_1_81	ERR540874	Scaffold: Velvet + Improvement	2826266	33	85644.42	327706	173972	6	156389	7	125434	9	108237	12	52187	16	399	33	784
12589_1_54	ERR540755	Scaffold: Velvet + Improvement	2918359	46	63442.59	300498	150835	7	124524	10	78741	12	60691	17	39096	22	422	46	3009
12593_1_82	ERR540875	Scaffold: Velvet + Improvement	2795891	22	127085.95	495758	175610	5	173963	6	131631	8	130700	10	108257	12	620	22	1646
12589_1_52	ERR540753	Scaffold: Velvet + Improvement	2758279	26	106087.65	731755	279248	3	156331	5	125425	7	110204	9	56801	13	447	26	690
12589_1_31	ERR540732	Scaffold: Velvet + Improvement	2790508	35	79728.8	325175	156338	6	125432	8	110659	11	79249	14	45581	18	446	35	1301
12589_1_32	ERR540733	Scaffold: Velvet + Improvement	2851671	30	95055.7	335438	170729	6	125457	8	119168	10	108282	13	66358	16	582	30	2188
12593_1_83	ERR540876	Scaffold: Velvet + Improvement	2739427	16	171214.19	642083	541567	3	541567	3	244004	4	178857	5	100069	8	693	16	1989
12589_1_35	ERR540736	Scaffold: Velvet + Improvement	2846771	30	94892.37	425231	211425	5	146367	7	116730	9	103808	11	54658	15	307	30	2316
12593_1_84	ERR540877	Scaffold: Velvet + Improvement	2772936	24	115539	680912	311055	3	183390	5	173971	6	115991	8	69232	11	397	24	2236
12593_1_47	ERR540840	Scaffold: Velvet + Improvement	2810386	41	68546	406668	152545	6	136208	8	125439	10	98904	13	77782	16	492	41	1439
12593_1_48	ERR540841	Scaffold: Velvet + Improvement	2785658	27	103172.52	375104	211785	5	173940	7	132019	9	110074	11	57910	14	653	27	792
12593_1_49	ERR540842	Scaffold: Velvet + Improvement	2813055	46	61153.37	254743	148162	8	125438	10	113438	12	88623	15	54709	19	647	46	778
12593_1_50	ERR540843	Scaffold: Velvet + Improvement	2815899	53	53130.17	365105	134203	7	108313	10	97537	12	55577	16	39826	22	399	53	1114
12593_1_51	ERR540844	Scaffold: Velvet + Improvement	2790352	28	99655.43	605640	364398	3	224812	4	174899	6	137484	7	61548	11	309	28	2062
12593_1_52	ERR540845	Scaffold: Velvet + Improvement	2787931	30	92931.03	348858	173985	6	157269	8	146344	10	125450	12	78623	15	307	30	2310
12593_1_53	ERR540846	Scaffold: Velvet + Improvement	2803380	14	200241.43	988098	603253	2	507635	3	507635	3	337127	4	130339	5	689	14	695
12593_1_86	ERR540879	Scaffold: Velvet + Improvement	2795672	15	186378.13	1024696	437005	2	351343	3	297089	4	170913	5	97519	7	445	15	3267
12593_1_87	ERR540880	Scaffold: Velvet + Improvement	2816771	16	176048.19	1339411	326299	2	315508	3	315508	3	285079	4	111642	6	391	16	4916
12589_1_55	ERR540756	Scaffold: Velvet + Improvement	2834425	28	101229.46	409607	211681	5	174565	6	146503	8	125431	10	55048	13	649	28	1065
12593_1_88	ERR540881	Scaffold: Velvet + Improvement	2791718	33	84597.52	333718	147001	6	124198	8	108234	11	75981	14	54602	18	440	33	529
12589_1_36	ERR540737	Scaffold: Velvet + Improvement	2859564	31	92244	448289	187790	5	171478	7	146506	8	89696	11	54468	15	444	31	722
12593_1_89	ERR540882	Scaffold: Velvet + Improvement	2799764	27	103694.96	297335	196766	6	156338	8	135462	10	108243	12	54665	16	1223	27	955
12593_1_90	ERR540883	Scaffold: Velvet + Improvement	2887116	49	58920.73	298267	190165	6	146366	8	125442	10	56802	13	38761	20	388	49	860
12593_1_91	ERR540884	Scaffold: Velvet + Improvement	2753909	14	196707.79	1230710	1004294	2	1004294	2	1004294	2	1004294	2	129792	4	470	14	2462
12589_1_53	ERR540754	Scaffold: Velvet + Improvement	2784925	15	185661.67	776653	325728	3	325728	3	241984	5	168023	6	118716	8	470	15	2637
12589_1_37	ERR540738	Scaffold: Velvet + Improvement	2841058	31	91647.03	409336	174028	5	146091	7	125443	9	111721	11	54514	14	626	31	815
12593_1_92	ERR540885	Scaffold: Velvet + Improvement	2766694	17	162746.71	1009743	718526	2	718526	2	222806	3	159849	5	129793	7	371	17	1942
12589_1_38	ERR540739	Scaffold: Velvet + Improvement	2829756	36	78604.33	450656	187587	5	130575	7	108353	10	95001	12	49094	17	309	36	672
12589_1_39	ERR540740	Scaffold: Velvet + Improvement	2827983	36	78555.08	297474	146751	7	125429	9	108591	11	87753	14	44578	19	399	36	932
12593_1_93	ERR540886	Scaffold: Velvet + Improvement	2907459	32	90858.09	433624	203342	5	170553	6	131426	8	119346	11	55058	14	363	32	1179
12589_1_40	ERR540741	Scaffold: Velvet + Improvement	2806788	28	100242.43	445379	214708	5	211378	6	183387	7	146398	9	108266	11	377	28	2286
12593_1_95	ERR540888	Scaffold: Velvet + Improvement	2879278	36	79979.94	340240	131082	6	110188	9	97707	11	57964	15	48978	20	367	36	488

12589_1_1	ERR540702	Scaffold: Velvet + Improvement	2778244	28	99223	626119	375215	3	174892	5	138538	6	90459	9	60209	13	306	28	1180
12589_1_2	ERR540703	Scaffold: Velvet + Improvement	2858688	51	56052.71	498406	126598	6	94953	9	79293	12	52701	17	36797	23	428	51	1562
12589_1_42	ERR540743	Scaffold: Velvet + Improvement	2742514	17	161324.35	732743	327301	3	327301	3	169260	5	163639	6	88299	9	472	17	3088
12589_1_43	ERR540744	Scaffold: Velvet + Improvement	2755754	21	131226.38	403326	236146	5	183610	6	158337	7	133089	9	108282	12	973	21	858
12589_1_44	ERR540745	Scaffold: Velvet + Improvement	2798775	30	93292.5	403038	215882	5	211583	6	147534	8	125425	10	106742	12	307	30	2114
12589_1_3	ERR540704	Scaffold: Velvet + Improvement	2769245	11	251749.55	1295238	705716	2	705716	2	705716	2	317477	3	108465	5	309	11	2405
12593_1_57	ERR540850	Scaffold: Velvet + Improvement	2754654	15	183643.6	601319	318708	4	318708	4	244035	5	174964	7	119789	9	482	15	2171
12589_1_45	ERR540746	Scaffold: Velvet + Improvement	2841530	33	86106.97	296737	173971	6	164080	8	125434	10	101514	13	57957	16	307	33	1242
12593_1_58	ERR540851	Scaffold: Velvet + Improvement	2814888	32	87965.25	534391	185703	4	128212	6	108233	9	95895	11	50740	16	649	32	518
12589_1_48	ERR540749	Scaffold: Velvet + Improvement	2943212	51	57710.04	252162	150923	8	145034	10	85106	13	59063	17	31497	24	318	51	5505
12589_1_47	ERR540748	Scaffold: Velvet + Improvement	2787172	29	96109.38	406980	190408	5	173942	7	125481	9	111738	11	54639	15	306	29	1393
12589_1_46	ERR540747	Scaffold: Velvet + Improvement	2796815	29	96441.9	402787	235908	5	174161	7	125433	9	119381	11	41135	14	440	29	1287
12589_1_4	ERR540705	Scaffold: Velvet + Improvement	2834862	26	109033.15	623869	158607	4	125431	6	119366	8	102221	11	75897	14	388	26	870
12589_1_5	ERR540706	Scaffold: Velvet + Improvement	2827121	30	94237.37	531558	214877	4	211419	5	146378	7	125436	9	54607	12	435	30	1479
12589_1_61	ERR540762	Scaffold: Velvet + Improvement	2786043	33	84425.55	317646	169740	6	169648	7	124399	10	90282	12	44029	16	709	33	2273
12593_1_59	ERR540852	Scaffold: Velvet + Improvement	2747569	27	101761.81	579132	187334	4	156340	6	139725	8	125434	10	56790	13	307	27	1149
12589_1_58	ERR540759	Scaffold: Velvet + Improvement	2847527	21	135596.52	858997	306322	3	209711	4	159283	6	134547	8	97518	10	431	21	1952
12589_1_59	ERR540760	Scaffold: Velvet + Improvement	2747354	12	228946.17	1292715	1000816	2	1000816	2	1000816	2	1000816	2	108440	4	395	12	2496
12593_1_61	ERR540854	Scaffold: Velvet + Improvement	2850051	45	63334.47	296316	148376	7	125456	9	95195	12	66440	15	41384	21	429	45	1710
12593_1_62	ERR540855	Scaffold: Velvet + Improvement	2766351	19	145597.42	931891	654638	2	169707	3	147396	5	129804	7	75795	9	371	19	3127
12593_1_63	ERR540856	Scaffold: Velvet + Improvement	2919428	46	63465.83	300521	155132	7	145777	9	103144	11	62658	15	39956	20	428	46	1711
12589_1_60	ERR540761	Scaffold: Velvet + Improvement	2737663	16	171103.94	775754	726968	2	333418	3	325588	4	108593	5	79187	8	346	16	3696
12589_1_65	ERR540766	Scaffold: Velvet + Improvement	2840257	18	157792.06	1048269	561329	2	381429	3	381429	3	379075	4	75154	6	564	18	1815
12589_1_66	ERR540767	Scaffold: Velvet + Improvement	2813103	17	165476.65	856984	811292	2	243734	3	223077	4	169614	5	129850	7	371	17	1865
12589_1_67	ERR540768	Scaffold: Velvet + Improvement	2937977	54	54406.98	298545	142108	8	124538	10	110095	12	66712	16	39068	22	338	54	2946
12589_1_68	ERR540769	Scaffold: Velvet + Improvement	2833910	32	88559.69	406835	215942	5	146511	7	110140	9	90194	12	54615	16	383	32	1228
12589_1_69	ERR540770	Scaffold: Velvet + Improvement	2788662	30	92955.4	581087	158445	5	148297	6	120706	9	97698	11	55342	15	307	30	721
12589_1_70	ERR540771	Scaffold: Velvet + Improvement	2892904	35	82654.4	426153	173965	6	171439	7	146604	9	68223	12	40099	17	628	35	2370
12589_1_71	ERR540772	Scaffold: Velvet + Improvement	2877622	36	79933.94	340067	177250	6	150860	8	146511	9	101172	12	44140	17	388	36	508
12589_1_72	ERR540773	Scaffold: Velvet + Improvement	2882132	44	65503	449078	174158	5	146511	7	111801	9	74074	12	38760	18	586	44	927
12589_1_73	ERR540774	Scaffold: Velvet + Improvement	2735694	14	195406.71	775721	723880	2	325343	3	180868	4	108680	6	79190	9	20494	14	1863
12589_1_74	ERR540775	Scaffold: Velvet + Improvement	2814049	14	201003.5	1339739	601644	2	601644	2	325875	3	325875	3	111646	5	383	14	2606
12755_8_71	ERR564227	Scaffold: Velvet + Improvement	2865761	34	84287.09	319522	162406	6	146536	8	125463	10	74230	13	47991	18	568	34	401
12755_8_72	ERR564228	Scaffold: Velvet + Improvement	2818206	50	56364.12	253105	149712	7	137355	9	108627	12	93564	14	41321	19	307	50	1038
12589_1_77	ERR540776	Scaffold: Velvet + Improvement	2828003	30	94266.77	354301	245290	5	169154	6	133322	8	112504	10	59648	14	307	30	169
12589_1_78	ERR540777	Scaffold: Velvet + Improvement	2805390	30	93513	562598	252894	4	236142	5	156330	7	125424	9	54686	12	393	30	1980
12589_1_80	ERR540779	Scaffold: Velvet + Improvement	2864831	38	75390.29	388148	174028	6	146518	8	108284	10	64654	13	42884	18	410	38	252
12589_1_81	ERR540780	Scaffold: Velvet + Improvement	2798011	32	87437.84	554442	254705	4	156333	6	125428	8	96956	10	42198	15	435	32	1068
12589_1_82	ERR540781	Scaffold: Velvet + Improvement	2810589	22	127754.05	406675	210561	5	183565	6	173966	8	146277	10	108254	12	451	22	846
12589_1_83	ERR540782	Scaffold: Velvet + Improvement	2859821	35	81709.17	424942	174158	5	146382	7	125425	9	94839	12	49351	16	383	35	1664
12589_1_84	ERR540783	Scaffold: Velvet + Improvement	2866437	35	81898.2	407023	173965	6	147926	7	139602	9	94895	12	41333	16	321	35	1382
12589_1_85	ERR540784	Scaffold: Velvet + Improvement	2860923	37	77322.24	433657	171480	5	147150	7	119347	9	114533	11	55087	15	405	37	845
12589_1_86	ERR540785	Scaffold: Velvet + Improvement	2818143	30	93938.1	578294	185011	5	134459	7	125317	9	123304	11	66958	14	440	30	1203
12589_1_87	ERR540786	Scaffold: Velvet + Improvement	2865549	33	86834.82	331730	204066	6	146394	8	125368	10	73544	13	40952	18	639	33	665
12589_1_88	ERR540787	Scaffold: Velvet + Improvement	2755369	21	131208.05	866011	211058	3	183277	4	174019	5	146406	7	108274	9	387	21	827
12593_2_77	ERR540964	Scaffold: Velvet + Improvement	2817766	27	104361.7	532248	215969	4	146997	6	125407	8	108235	10	58332	14	429	27	1063
12589_1_89	ERR540788	Scaffold: Velvet + Improvement	2858637	39	73298.38	310875	173809	6	156339	8	125441	10	88637	13	40099	18	388	39	1619
12755_8_73	ERR564229	Scaffold: Velvet + Improvement	2845498	32	88921.81	406501	174013	5	154360	7	125438	9	104834	12	39384	16	307	32	508
12589_1_90	ERR540789	Scaffold: Velvet + Improvement	2874947	24	119789.46	506689	298007	4	285170	5	145664	7	103514	9	85830	12	637	24	2080
12589_1_91	ERR540790	Scaffold: Velvet + Improvement	2842482	27	105277.11	310544	181666	6	146335	8	131609	10	108253	12	54472	17	651	27	1277
12589_1_92	ERR540791	Scaffold: Velvet + Improvement	2864377	39	73445.56	335288	146496	6	125425	8	122075	10	92343	13	42587	18	325	39	742
12589_1_94	ERR540793	Scaffold: Velvet + Improvement	2810815	41	68556.46	311002	146329	7	125428	9	111241	11	67141	14	48310	19	327	41	1642
12593_2_78	ERR540965	Scaffold: Velvet + Improvement	2757664	24	114902.67	578259	183012	4	170563	6	155413	7	125425	9	69230	12	388	24	521
12589_1_95	ERR540794	Scaffold: Velvet + Improvement	2746902	30	91563.4	586489	187386	4	156334	6	139125	8	125434	10	57962	12	440	30	564
12593_2_1	ERR540889	Scaffold: Velvet + Improvement	2784280	23	121055.65	435719	210501	5	176881	6	156383	8	108243	10	62596	13	390	23	1118
12593_2_2	ERR540890	Scaffold: Velvet + Improvement	2884506	37	77959.62	336366	159531	6	133173	8	108329	11	77237	14	41132	19	423	37	852
12593_2_3	ERR540891	Scaffold: Velvet + Improvement	2869644	29	98953.24	342907	211791	5	174001	7	125430	9	98213	11	48309	15	307	29	1844
12593_2_7	ERR540895	Scaffold: Velvet + Improvement	2736057	14	195432.64	674073	394939	3	286788	4	225102	5	187060	6	87900	8	606	14	2859
12593_2_8	ERR540896	Scaffold: Velvet + Improvement	2822279	28	100795.68	923034	592632	2	243625	3	221642	4	119028	7	86028	9	482	28	2094
12593_2_9	ERR540897	Scaffold: Velvet + Improvement	2783486	29	95982.28	403337	156332	5	146342	7	125624	9	89505	12	57962	15	598	29	515
12593_2_10	ERR540898	Scaffold: Velvet + Improvement	2796401	27	103570.41	432312	209690	5	170588	7	146373	8	111794	11	57902	14	307	27	719
12593_2_11	ERR540899	Scaffold: Velvet + Improvement	2734852	26	105186.62	601579	209205	4	135752	6	96638	8	81387	12	64855	15	3475	26	1254
12593_2_12	ERR540900	Scaffold: Velvet + Improvement	2850371	31	91947.45	447811	236605	4	185649	6	173972	7	108277	10	88614	12	633	31	1981
12593_2_13	ERR540901	Scaffold: Velvet + Improvement	2873404	33	87072.85	308426	167280	7	167181	8	108598	11	102642	13	56802	17	307	33	586
12593_2_14	ERR540902	Scaffold: Velvet + Improvement	2875063	33	87123.12	391787	167183	6	131604	8	124635	10	108254	13	41130	17	387	33	1619
12593_2_15	ERR540903	Scaffold: Velvet + Improvement	2796480	14	199748.57	1276109	393426	2	387104	3	387104	3	147934	5	92525	7	945	14	2501
12593_2_16	ERR540904	Scaffold: Velvet + Improvement	2806264	24	116927.67	584728	190438	5	176875	6	128264	8	125415	10	67011	13	651	24	1152

12593_2_17	ERR540905	Scaffold: Velvet + Improvement	2826252	47	60133.02	432174	125425	7	119813	9	87298	12	73948	15	48775	20	307	47	801
12593_2_18	ERR540906	Scaffold: Velvet + Improvement	2832323	34	83303.62	402665	173975	6	170554	7	131568	9	89676	12	42859	16	423	34	178
12593_2_20	ERR540908	Scaffold: Velvet + Improvement	2913436	41	71059.41	384115	224535	6	175744	7	145795	9	78743	12	42534	16	313	41	1387
12593_2_21	ERR540909	Scaffold: Velvet + Improvement	2841399	33	86103	311221	155433	6	131541	8	110179	11	90570	13	48102	18	307	33	931
12593_2_22	ERR540910	Scaffold: Velvet + Improvement	2793063	15	186204.2	783665	327586	3	263175	4	207023	5	202464	6	118847	8	516	15	2431
12593_2_25	ERR540913	Scaffold: Velvet + Improvement	2808505	31	90596.94	449202	252896	5	236643	6	146266	7	101026	10	57932	13	440	31	1106
12593_2_79	ERR540966	Scaffold: Velvet + Improvement	2859996	34	84117.53	311794	156383	7	125428	9	108244	11	79972	14	55195	18	442	34	1360
12593_2_26	ERR540914	Scaffold: Velvet + Improvement	2844388	35	81268.23	335264	180716	6	176874	7	117398	10	91814	12	42657	17	598	35	618
12593_2_27	ERR540915	Scaffold: Velvet + Improvement	2798768	31	90282.84	339954	145480	7	125452	9	108343	11	90224	14	54704	19	307	31	712
12593_2_80	ERR540967	Scaffold: Velvet + Improvement	2776565	15	185104.33	936644	405202	3	405202	3	319628	4	279326	5	212622	6	310	15	3050
12593_2_28	ERR540916	Scaffold: Velvet + Improvement	2877882	43	66927.49	493057	222073	4	221206	5	159380	7	122996	9	88498	11	395	43	3751
12593_2_74	ERR540961	Scaffold: Velvet + Improvement	2755223	26	105970.12	537138	270494	4	173963	6	170562	7	125434	9	64819	12	440	26	887
12593_2_29	ERR540917	Scaffold: Velvet + Improvement	2754003	24	114750.12	578231	198486	4	156381	6	130679	8	111745	10	90532	13	630	24	612
12593_2_30	ERR540918	Scaffold: Velvet + Improvement	2773695	33	84051.36	380518	172455	6	142660	8	137078	10	87780	13	56361	17	338	33	1763
12593_2_31	ERR540919	Scaffold: Velvet + Improvement	2809265	26	108048.65	449758	198563	5	166220	7	152687	9	146107	10	73400	13	307	26	1688
12593_2_34	ERR540922	Scaffold: Velvet + Improvement	2853024	38	75079.58	359249	156334	6	125425	8	112754	10	68741	13	39379	19	391	38	1294
12593_2_40	ERR540927	Scaffold: Velvet + Improvement	2735446	16	170965.38	775746	721678	2	378770	3	325222	4	325222	4	88131	7	336	16	2658
12593_2_42	ERR540929	Scaffold: Velvet + Improvement	2810521	33	85167.3	532086	253199	4	156377	6	133906	8	111264	10	44626	15	307	33	2043
12593_2_43	ERR540930	Scaffold: Velvet + Improvement	2866408	33	86860.85	452294	198397	5	171443	7	123044	9	88613	12	54700	16	451	33	835
12593_2_37	ERR540925	Scaffold: Velvet + Improvement	2787135	34	81974.56	313355	156269	7	139517	9	125425	11	87412	14	54639	18	401	34	946
12593_2_38	ERR540926	Scaffold: Velvet + Improvement	2781323	23	120927.09	424252	288853	4	282196	5	237034	7	135762	8	108253	10	450	23	1037
12593_2_45	ERR540932	Scaffold: Velvet + Improvement	2858091	24	119087.12	889981	347903	3	304741	4	210610	5	173083	6	97536	9	414	24	2947
12593_2_46	ERR540933	Scaffold: Velvet + Improvement	2854137	39	73183	408720	174028	6	146349	8	119365	10	65849	13	38821	19	307	39	2005
12593_2_47	ERR540934	Scaffold: Velvet + Improvement	2747192	25	109887.68	531601	429664	3	406700	4	146336	5	120417	7	113621	9	307	25	1008
12593_2_48	ERR540935	Scaffold: Velvet + Improvement	2840984	44	64567.82	325722	173981	7	131972	8	86863	11	67139	15	54697	19	329	44	2353
12593_2_49	ERR540936	Scaffold: Velvet + Improvement	2782682	25	111307.28	389949	180469	6	158795	8	146330	10	125365	12	65530	14	399	25	779
12593_2_50	ERR540937	Scaffold: Velvet + Improvement	2827626	29	97504.34	449052	161765	6	156332	7	132028	9	111723	12	57961	15	975	29	343
12593_2_51	ERR540938	Scaffold: Velvet + Improvement	2760287	9	306698.56	1170217	1018563	2	1018563	2	1018563	2	243954	3	168520	4	2756	9	1206
12593_2_52	ERR540939	Scaffold: Velvet + Improvement	2783598	30	92786.6	371722	209323	5	173921	7	156341	8	125432	10	50978	14	385	30	696
12593_2_53	ERR540940	Scaffold: Velvet + Improvement	2856170	38	75162.37	332380	146487	6	125441	8	94986	11	73766	14	51966	19	307	38	1861
12593_2_54	ERR540941	Scaffold: Velvet + Improvement	2907721	52	55917.71	325147	172474	6	133757	8	92608	11	65108	14	38757	20	302	52	1944
12593_2_55	ERR540942	Scaffold: Velvet + Improvement	2793753	23	121467.52	891425	227118	3	183228	5	174864	6	137484	8	66086	11	392	23	2056
12593_2_56	ERR540943	Scaffold: Velvet + Improvement	2852512	35	81500.34	506997	170975	5	150844	6	132433	8	76384	12	56169	16	421	35	1790
12593_2_81	ERR540968	Scaffold: Velvet + Improvement	2694936	24	112289	399842	169681	5	168385	6	128047	8	90290	11	44035	15	7047	24	1787
12593_2_61	ERR540948	Scaffold: Velvet + Improvement	2807408	33	85072.97	340311	174029	6	170595	7	146425	9	94914	12	57674	15	392	33	2302
12593_2_62	ERR540949	Scaffold: Velvet + Improvement	2928689	32	91521.53	611252	311090	4	298745	5	150968	6	86607	9	55767	12	314	32	2375
12593_2_82	ERR540969	Scaffold: Velvet + Improvement	2819094	27	104410.89	428301	185704	5	170664	7	127221	9	108242	11	54534	15	632	27	1720
12593_2_64	ERR540951	Scaffold: Velvet + Improvement	2800241	17	164720.06	711581	355018	3	338822	4	226518	5	200392	6	95814	8	330	17	2707
12593_2_57	ERR540944	Scaffold: Velvet + Improvement	2731988	12	227665.67	1091846	771520	2	771520	2	452614	3	452614	3	81946	5	371	12	2366
12593_2_58	ERR540945	Scaffold: Velvet + Improvement	2797301	30	93243.37	571361	210567	4	170551	6	134958	8	123341	10	57966	13	307	30	1129
12593_2_59	ERR540946	Scaffold: Velvet + Improvement	2828444	23	122975.83	1237435	426260	2	353575	3	353575	3	108618	5	50807	9	446	23	2664
12593_2_60	ERR540947	Scaffold: Velvet + Improvement	2791233	39	71570.08	289393	172432	7	139850	9	123654	11	108242	13	61287	17	306	39	1046
12593_2_69	ERR540956	Scaffold: Velvet + Improvement	2774566	31	89502.13	357087	238007	5	156331	7	125421	9	110203	11	69285	14	415	31	952
12593_2_70	ERR540957	Scaffold: Velvet + Improvement	2798601	30	93286.7	339941	176880	6	174619	7	131414	9	93410	12	65147	15	307	30	1929
12593_2_71	ERR540958	Scaffold: Velvet + Improvement	2886555	37	78015	338798	209770	5	173960	7	147929	9	113407	11	43999	15	387	37	0
12593_2_83	ERR540970	Scaffold: Velvet + Improvement	2734638	18	151924.33	766419	726482	2	277567	3	243733	4	134247	6	88806	8	794	18	2569
12593_2_72	ERR540959	Scaffold: Velvet + Improvement	2823402	33	85557.64	455215	167361	5	156379	6	128263	8	108269	11	54681	14	430	33	1204
12593_2_65	ERR540952	Scaffold: Velvet + Improvement	2785432	27	103164.15	446139	212973	5	183457	6	146365	8	125432	10	87734	13	307	27	1392
12593_2_84	ERR540971	Scaffold: Velvet + Improvement	2734798	13	210369.08	889306	721618	2	378876	3	378876	3	325411	4	79196	6	371	13	2247
12593_2_66	ERR540953	Scaffold: Velvet + Improvement	2779769	31	89669.97	693654	331984	3	224831	4	137476	6	92947	9	67739	12	311	31	1778
12593_2_67	ERR540954	Scaffold: Velvet + Improvement	2778851	13	213757.77	1287747	458670	2	458670	2	317396	3	225968	4	108953	6	560	13	1784
12593_2_68	ERR540955	Scaffold: Velvet + Improvement	2950686	28	105381.64	714737	263489	4	256582	5	154734	6	114520	8	54411	12	1236	28	1038
12593_2_91	ERR540978	Scaffold: Velvet + Improvement	2902287	44	65961.07	252201	150384	8	147684	9	85227	12	60692	16	41527	21	399	44	2988
12593_2_92	ERR540979	Scaffold: Velvet + Improvement	2784102	31	89809.74	404303	210634	5	173999	6	146373	8	111640	10	50888	14	391	31	976
12593_2_93	ERR540980	Scaffold: Velvet + Improvement	2762776	35	78936.46	581322	147926	6	131468	8	125429	10	76685	13	46075	17	442	35	1023
12593_2_95	ERR540982	Scaffold: Velvet + Improvement	2861195	16	178824.69	1418386	542339	2	542339	2	354857	3	354857	3	108484	5	390	16	4522
12673_8_44	ERR555043	Scaffold: Velvet + Improvement	2878255	18	159903.06	864716	366118	3	361050	4	211146	5	173195	6	107222	8	380	18	2417
12673_8_78	ERR555077	Scaffold: Velvet + Improvement	2847565	36	79099.03	310572	158375	7	125422	9	114371	11	83031	14	54694	18	459	36	888
12673_8_60	ERR555059	Scaffold: Velvet + Improvement	2882444	22	131020.18	1010231	336319	3	336319	3	309847	4	159067	6	59817	9	420	22	2542
12673_8_61	ERR555060	Scaffold: Velvet + Improvement	2786185	23	121138.48	670162	352485	3	335818	4	307279	5	200290	6	53223	10	353	23	2737
12673_8_70	ERR555069	Scaffold: Velvet + Improvement	2952480	37	79796.76	714882	256733	4	155294	6	151280	8	114666	10	48633	15	442	37	3325
12673_8_71	ERR555070	Scaffold: Velvet + Improvement	2876933	33	87179.79	450850	173973	5	146622	7	108671	10	103554	12	54997	16	333	33	1690
12673_8_77	ERR555076	Scaffold: Velvet + Improvement	2898647	29	99953.34	611081	367375	4	367375	4	314445	5	129632	7	78536	10	410	29	1776
12673_8_62	ERR555061	Scaffold: Velvet + Improvement	2915191	33	88339.12	464350	314988	4	284782	5	145823	7	106200	9	55742	12	348	33	1467
12673_8_63	ERR555062	Scaffold: Velvet + Improvement	2772052	12	231004.33	1291631	543624	2	543624	2	482603	3	482603	3	108487	5	330	12	1170
12673_8_66	ERR555065	Scaffold: Velvet + Improvement	2776146	17	163302.71	1090666	775789	2	775789	2	380202	3	380202	3	68099	7	371	17	2801
12673_8_55	ERR555054	Scaffold: Velvet + Improvement	2769117	19	145743	387472	240923	5	222630	6	189860	7	131274	9	118404	11	490	19	1176

12673_8_56	ERR555055	Scaffold: Velvet + Improvement	2827916	44	64270.82	338338	158318	7	146445	8	125438	10	84648	13	39380	18	444	44	744
12673_8_68	ERR555067	Scaffold: Velvet + Improvement	2882836	29	99408.14	505603	211932	4	154058	6	135768	7	97815	10	65984	14	473	29	1109
12673_8_69	ERR555068	Scaffold: Velvet + Improvement	2798393	25	111935.72	409004	190333	5	170555	7	144132	9	124509	11	63334	14	620	25	272
12673_8_76	ERR555075	Scaffold: Velvet + Improvement	2813559	60	46892.65	277173	156412	7	110270	9	104733	12	68211	15	37114	21	307	60	851
12673_8_51	ERR555050	Scaffold: Velvet + Improvement	2816484	9	312942.67	1335735	544265	2	544265	2	531138	3	531138	3	172866	4	2572	9	2231
12673_8_52	ERR555051	Scaffold: Velvet + Improvement	2778989	35	79399.69	400703	174169	6	128392	8	124645	10	87191	12	43578	17	442	35	810
12673_8_79	ERR555078	Scaffold: Velvet + Improvement	2798664	42	66634.86	338698	125425	7	108274	10	98432	12	62772	16	41997	21	313	42	877
12673_8_80	ERR555079	Scaffold: Velvet + Improvement	2746006	14	196143.29	971061	584611	2	382603	3	382603	3	353652	4	92327	6	428	14	1557
12673_8_81	ERR555080	Scaffold: Velvet + Improvement	2825296	25	113011.84	893943	255709	3	243669	4	193002	5	121930	7	89854	9	368	25	889
12673_8_54	ERR555053	Scaffold: Velvet + Improvement	2772753	13	213288.69	1291764	428796	2	428796	2	425606	3	226553	4	172833	5	396	13	2265
12673_8_57	ERR555056	Scaffold: Velvet + Improvement	2833486	27	104943.93	406858	211675	5	170559	7	146549	8	108285	11	54696	14	444	27	1325
12673_8_58	ERR555057	Scaffold: Velvet + Improvement	2737122	16	171070.12	766047	385595	3	325538	4	325538	4	143355	6	90260	8	371	16	308
12673_8_59	ERR555058	Scaffold: Velvet + Improvement	2710636	16	169414.75	1299005	625334	2	625334	2	625334	2	140463	4	108559	6	600	16	519
12593_2_85	ERR540972	Scaffold: Velvet + Improvement	2759201	28	98542.89	654828	174012	4	170550	5	139575	7	108885	10	59043	13	307	28	1299
12673_8_45	ERR555044	Scaffold: Velvet + Improvement	2844311	35	81266.03	330651	166819	6	139476	8	125430	11	108902	13	52184	16	410	35	961
12673_8_46	ERR555045	Scaffold: Velvet + Improvement	2990078	66	45304.21	291353	111034	10	77709	14	65134	18	49263	23	29498	30	305	66	2445
12673_8_48	ERR555047	Scaffold: Velvet + Improvement	2786913	26	107188.96	446091	245293	4	176881	6	173986	7	130633	9	108236	11	516	26	3
12673_8_47	ERR555046	Scaffold: Velvet + Improvement	2927433	34	86100.97	782091	298630	4	284707	5	129618	6	99154	9	86607	12	359	34	1166
12673_8_50	ERR555049	Scaffold: Velvet + Improvement	2912386	46	63312.74	304400	150843	7	127663	9	98669	12	78495	15	39091	21	394	46	1425
12673_8_53	ERR555052	Scaffold: Velvet + Improvement	2810797	54	52051.8	254996	120153	9	109656	11	91166	14	56704	18	39253	24	376	54	1615
12673_8_72	ERR555071	Scaffold: Velvet + Improvement	2830164	43	65817.77	311105	146329	7	125426	9	108274	11	65519	15	43108	21	361	43	908
12673_8_73	ERR555072	Scaffold: Velvet + Improvement	2786692	31	89893.29	444390	209377	5	156360	7	146334	8	108243	11	55731	14	447	31	974
12673_8_74	ERR555073	Scaffold: Velvet + Improvement	2770516	36	76958.78	399519	134226	7	113872	9	96684	12	75391	15	50236	19	361	36	2445
12593_2_87	ERR540974	Scaffold: Velvet + Improvement	2748159	26	105698.42	591719	174014	4	170566	5	131306	7	109105	9	87402	12	389	26	388
12593_2_88	ERR540975	Scaffold: Velvet + Improvement	2863268	36	79535.22	386184	190191	5	156332	7	125427	9	67355	12	41126	17	439	36	1502
12593_2_89	ERR540976	Scaffold: Velvet + Improvement	2890755	42	68827.5	335252	255227	5	146380	7	108297	10	55054	13	41236	19	400	42	948
12593_2_90	ERR540977	Scaffold: Velvet + Improvement	2867185	39	73517.56	300290	170885	7	150885	8	85103	11	67664	14	50101	19	421	39	2102
12593_2_86	ERR540973	Scaffold: Velvet + Improvement	2830337	36	78620.47	406473	168691	6	135478	8	108184	10	67016	13	41321	18	398	36	831
