# Supplementary material for: Prospective genomic surveillance of methicillin-resistant Staphylococcus aureus (MRSA) associated with bloodstream infection, England, 1 October 2012 to 30 September 2013
Source: Euro Surveill. 2019 Jan 24;24(4):1800215. doi: 10.2807/1560-7917.ES.2019.24.4.1800215 (PMC6351993; doi:10.2807/1560-7917.ES.2019.24.4.1800215)
Supplement: Supplementary Table S2 [file 1800215_TOLEMAN_SupplementaryTableS2.pdf]

Supplementary Table S2: Combined genomic and epidemiological data.

This supplementary material is hosted by Eurosurveillance as supporting information alongside the article 'Prospective genomic surveillance of methicillin-resistant *Staphylococcus aureus* (MRSA) associated with bloodstream infection, England, 1 October 2012 to 30 September 2013' on behalf of the authors who remain responsible for the accuracy and appropriateness of the content.
The same standards for ethics, copyright, attributions and permissions as for the article apply. Eurosurveillance is not responsible for the maintenance of any links or email addresses provided therein.

**Sample Details**					**Laboratory Data**				
**Strain ID**	**Year of Isolation**	**CC (genomic)**	**ST (genomic)**	**spa (genomic)**	**mecA**	**PVL**	**spa**	**Region**	**Community or Hospital onset**
12483_8_74	2012	22	22	t608	1	0	t608	EAST	HO
12483_8_17	2012	5	5	t032	1	0	t032	S EAST	HO
12625_1_22	2012	5	5	t548	1	0	t548	YORK&HUM	CO
12483_8_71	2012	22	22	t032	1	0	t032	W MIDS	HO
12625_1_23	2012	22	3742	t906	1	0	t906	LONDON	HO
12625_1_20	2012	22	22	t032	1	0	t032	YORK&HUM	CO
12625_1_17	2012	22	22	t032	1	0	t032	LONDON	HO
12483_8_73	2012	5	125	t067	1	0	t067	LONDON	NK
12483_8_69	2012	8	8	t121	1	1	t121	EAST	CO
12483_8_61	2012	22	22	t032	1	0	t032	S EAST	CO
12625_1_24	2012	22	22	t005	1	1	t005	N WEST	HO
12625_1_25	2012	30	36	t1820	1	0	t1820	N WEST	HO
12625_1_13	2012	22	22	t022	1	0	t022	YORK&HUM	HO
12483_8_45	2012	22	22	t10125	1	0	t10125	N WEST	HO
12483_8_31	2012	22	22	t2892	1	0	t2892	S EAST	NK
12483_8_68	2012	22	22	t032	1	0	t032	S WEST	NK
12483_8_47	2012	30	36	t268	1	0	t268	NK	NK
12483_8_21	2012	22	22	t432	1	0	t432	LONDON	CO
12483_8_30	2012	22	22	t032	1	0	t032	N WEST	NK
12483_8_78	2012	22	22	t032	1	0	t032	S EAST	NK
12483_8_20	2012	22	22	t032	1	0	t032	S EAST	HO
12483_8_19	2012	22	22	t022	1	0	t022	LONDON	HO
12673_8_85	2012	22	22	t790	1	0	t790	LONDON	HO
12625_1_26	2012	30	36	t018	1	0	t018	S EAST	HO
12625_1_18	2012	22	22	t032	1	0	t032	LONDON	CO
12625_1_29	2012	22	22	t032	1	0	t032	N WEST	CO
12483_8_77	2012	22	22	t032	1	0	t032	N WEST	HO
12625_1_11	2012	59	59	t316	1	0	t316	W MIDS	HO
12625_1_8	2012	22	22	t379	1	0	t379	S EAST	HO
12625_1_19	2012	22	22	t032	1	0	t032	EAST	CO
12483_8_49	2012	22	22	t032	1	0	t032	EAST	CO
12483_8_58	2012	22	22	t032	1	0	t032	EAST	CO
12483_8_35	2012	22	22	t005	1	1	t005	LONDON	CO
12483_8_75	2012	22	22	t032	1	0	t032	S WEST	HO
12673_8_86	2012	8	94	t008	1	0	t008	LONDON	CO
12483_8_25	2012	22	22	t032	1	0	t032	N EAST	CO
12483_8_24	2012	22	22	t032	1	0	t032	S EAST	HO
12625_1_9	2012	22	22	t032	1	0	t032	S WEST	CO
12625_1_10	2012	12	12	t160	1	0	t160	S WEST	NK

12673_8_87	2012	8	8	t008	1	0	t008	LONDON	HO
12483_8_65	2012	59	59	t316	1	0	t316	YORK&HUM	HO
12625_1_5	2012	22	22	t1218	1	0	t1218	N WEST	CO
12483_8_76	2012	22	22	t032	1	0	t032	S EAST	HO
12483_8_87	2012	22	22	t032	1	0	t032	NK	NK
12483_8_88	2012	5	5	t032	1	0	t032	LONDON	CO
12483_8_93	2012	8	8	t008	1	0	t008	LONDON	HO
12483_8_2	2012	22	22	t785	1	0	t785	YORK&HUM	CO
12483_8_52	2012	22	22	t032	1	0	t032	NK	NK
12483_8_83	2012	22	22	t032	1	0	t032	E MIDS	HO
12673_8_88	2012	45	45	t032	1	0	t032	S WEST	HO
12483_8_28	2012	22	22	t906	1	0	t906	N WEST	HO
12625_1_14	2012	22	22	t032	1	0	t032	S EAST	HO
12625_1_3	2012	1	1	t127	1	0	t127	N WEST	CO
12483_8_16	2012	22	22	t032	1	0	t032	NK	NK
12483_8_55	2012	22	22	t032	1	0	t032	S EAST	HO
12483_8_81	2012	30	36	t018	1	0	t018	LONDON	HO
12625_1_27	2012	22	22	t032	1	0	t032	N EAST	HO
12483_8_51	2012	30	30	t1749	1	1	t1749	YORK&HUM	HO
12483_8_4	2012	22	22	t513	1	0	t513	S WEST	NK
12483_8_56	2012	22	22	- (Deletions/rearrangements within the spa gene. Loss of sequence complementary to forward primer)	1	0	t032	W MIDS	CO
12483_8_32	2012	22	22	t2818	1	0	t2818	S WEST	CO
12483_8_92	2012	22	22	t578	1	0	t578	W MIDS	CO
12483_8_54	2012	22	22	t032	1	0	t032	S EAST	CO
12483_8_95	2012	22	22	t10172	1	0	t10172	S WEST	CO
12483_8_59	2012	22	22	t148	1	0	t148	W MIDS	CO
12483_8_1	2012	22	22	t032	1	0	t032	N WEST	CO
12483_8_11	2012	22	22	t022	1	0	t022	N WEST	HO
12625_1_2	2012	22	22	t032	1	0	t032	LONDON	HO
12625_1_15	2012	22	22	t032	1	0	t032	LONDON	HO
12483_8_41	2012	22	22	t1370	1	0	t1370	LONDON	HO
12483_8_60	2012	unknown_CC	72	t032	1	0	t032	LONDON	HO
12483_8_62	2012	22	22	t032	1	0	t032	NK	NK
12625_1_7	2012	22	22	t032	1	0	t032	LONDON	CO
12483_8_15	2012	5	105	t045	1	0	t045	S WEST	HO
12483_8_7	2012	22	22	t022	1	0	t022	S WEST	NK
12625_1_4	2012	22	22	t4929	1	0	t4929	S WEST	NK
12483_8_80	2012	5	5	t6445	1	0	t6445	W MIDS	NK
12483_8_66	2012	30	30	t019	1	1	t019	LONDON	CO
12483_8_90	2012	5	5	t002	1	0	t002	S WEST	NK
12483_8_53	2012	22	22	t4218	1	0	t4218	S WEST	NK
12483_8_34	2012	22	22	t032	1	0	t032	LONDON	HO
12625_1_1	2012	22	22	t020	1	0	t020	W MIDS	HO
12483_8_86	2012	30	36	t018	1	0	t018	LONDON	HO
12483_8_79	2012	59	3669	t529	1	0	t529	S WEST	NK

12483_8_9	2012	22	22	t2857	1	0	t2857	W MIDS	NK
12483_8_14	2012	59	59	t316	1	0	t316	YORK&HUM	HO
12483_8_63	2012	5	5	t311	1	0	t311	EAST	CO
12483_8_29	2012	30	36	t018	1	0	t018	S WEST	HO
12483_8_82	2012	22	22	t022	1	0	t022	LONDON	HO
12483_8_3	2012	30	36	t253	1	0	t253	E MIDS	CO
12483_8_57	2012	22	22	t032	1	0	t032	E MIDS	CO
12483_8_38	2012	22	22	t032	1	0	t032	YORK&HUM	HO
12483_8_42	2012	22	22	t005	1	1	t005	LONDON	NK
12483_8_36	2012	22	22	t005	1	0	t005	YORK&HUM	HO
12483_8_67	2012	22	22	t032	1	0	t032	S EAST	CO
12625_1_28	2012	22	22	t032	1	0	t032	N WEST	HO
12483_8_50	2012	22	22	t022	1	0	t022	YORK&HUM	HO
12483_8_89	2012	5	5	t010	1	0	t010	E MIDS	CO
12625_1_6	2012	30	30	t019	1	1	t019	S EAST	CO
12755_8_68	2012	1	1	t127	1	1	t127	N WEST	CO
12483_8_94	2012	22	22	t032	1	0	t032	NK	NK
12483_8_84	2012	22	22	t020	1	0	t020	N WEST	NK
12483_8_26	2012	8	3727	t334	1	0	t334	S WEST	NK
12625_1_30	2012	30	36	t018	1	0	t018	LONDON	HO
12625_1_32	2012	5	3743	t1781	1	0	t1781	N WEST	CO
12589_1_6	2012	22	22	t3612	1	0	t3612	S WEST	CO
12625_1_33	2012	22	22	t020	1	0	t020	S WEST	CO
12625_1_34	2012	5	526	- (Deletions/rearrangements within the spa gene. Loss of sequence complementary to forward primer)	1	1	t002	N WEST	CO
12625_1_35	2012	22	22	t032	1	0	t032	EAST	HO
12625_1_36	2012	22	22	t032	1	0	t032	N EAST	NK
12625_1_37	2012	22	22	t032	1	0	t032	N EAST	NK
12625_1_38	2012	88	88	t1816	1	1	t1816	S EAST	NK
12625_1_42	2012	22	22	t8530	1	0	t8530	S WEST	CO
12625_1_43	2012	1	1	t127	1	0	t127	LONDON	HO
12625_1_45	2012	22	22	t11666	1	0	t11666	S WEST	CO
12625_1_46	2012	22	22	t3612	1	0	t3612	S WEST	CO
12625_1_47	2012	22	22	t022	1	0	t022	S WEST	CO
12625_1_48	2012	22	22	t432	1	0	t432	W MIDS	CO
12625_1_49	2012	22	22	t4573	1	1	t4573	W MIDS	CO
12625_1_53	2013	22	3122	t032	1	0	t032	S EAST	HO
12625_1_54	2013	22	22	t032	1	0	t032	S EAST	HO
12625_1_50	2012	22	22	t032	1	0	t032	N EAST	HO
12625_1_51	2013	22	22	t032	1	0	t032	W MIDS	CO
12625_1_52	2013	22	22	t032	1	0	t032	W MIDS	CO
12625_1_55	2013	8	8	t104	1	1	t104	S EAST	NK
12625_1_56	2012	22	22	t1041	1	0	t1041	EAST	CO
12625_1_57	2013	22	22	t8473	1	0	t8473	LONDON	HO
12625_1_59	2013	22	22	t032	1	0	t032	S EAST	HO
12625_1_60	2013	22	22	t032	1	0	t032	LONDON	HO

12625_1_61	2013	22	22	t032	1	0	t032	YORK&HUM	CO
12625_1_62	2013	22	22	t032	1	0	t032	LONDON	CO
12625_1_63	2012	22	22	t032	1	0	t032	N EAST	NK
12625_1_64	2013	22	22	t022	1	0	t022	LONDON	HO
12625_1_65	2013	unknown_CC	80	t044	1	1	t044	NK	NK
12589_1_7	2012	22	22	t032	1	0	t032	E MIDS	CO
12625_1_66	2013	22	22	t032	1	0	t032	E MIDS	HO
12625_1_67	2013	30	36	t018	1	0	t018	N WEST	HO
12625_1_69	2013	22	22	t032	1	0	t032	S EAST	CO
12625_1_70	2013	22	22	t906	1	0	t906	N WEST	CO
12625_1_71	2013	22	22	t1499	1	0	t1499	N WEST	NK
12625_1_72	2013	1	1	t127	1	0	t127	EAST	CO
12625_1_73	2013	22	22	t032	1	0	t032	S EAST	HO
12625_1_74	2013	22	22	t032	1	0	t032	LONDON	HO
12625_1_75	2013	8	8	t008	1	1	t008	YORK&HUM	CO
12625_1_76	2013	45	46	t040	1	0	t040	EAST	CO
12625_1_78	2013	unknown_CC	93	t202	1	1	t202	S EAST	CO
12625_1_79	2013	30	3674	t253	1	0	t253	E MIDS	HO
12625_1_80	2013	1	772	t657	1	1	t657	S WEST	NK
12625_1_81	2013	5	5	t002	1	1	t002	LONDON	HO
12625_1_82	2013	30	36	t018	1	0	t018	N WEST	CO
12625_1_83	2013	22	22	t1612	1	0	t1612	S WEST	NK
12625_1_84	2013	22	22	t032	1	0	t032	LONDON	CO
12625_1_85	2013	22	22	t032	1	0	t032	NK	NK
12625_1_86	2013	45	45	t077	1	0	t077	LONDON	CO
12625_1_87	2013	5	5	t002	1	1	t002	LONDON	HO
12625_1_89	2013	22	22	t032	1	0	t032	N WEST	CO
12625_1_90	2013	22	22	t852	1	1	t852	N WEST	HO
12625_1_91	2013	22	22	t032	1	0	t032	N WEST	CO
12625_1_92	2013	22	22	t032	1	0	t032	S EAST	HO
12625_1_93	2013	22	22	t020	1	0	t020	S EAST	CO
12625_1_95	2013	22	22	t032	1	0	t032	EAST	CO
12593_1_1	2013	5	5	t2724	1	0	t2724	N WEST	CO
12593_1_2	2013	22	22	t032	1	0	t032	S EAST	CO
12593_1_4	2013	22	22	t11885	1	0	t11885	LONDON	HO
12593_1_64	2013	5	5	t002	1	0	t002	LONDON	CO
12593_1_5	2013	22	22	t032	1	0	t032	E MIDS	CO
12593_1_6	2013	30	36	t018	1	0	t018	LONDON	HO
12593_1_7	2013	22	22	t11279	1	0	t11279	S EAST	NK
12593_1_9	2013	22	22	t020	1	0	t020	EAST	HO
12593_1_10	2013	1	1	t127	1	0	t127	N WEST	CO
12593_1_11	2013	22	22	t032	1	0	t032	N WEST	HO
12593_1_13	2013	22	22	t8964	1	0	t8964	EAST	CO
12593_1_65	2013	8	239	t037	1	0	t037	EAST	HO
12593_1_15	2013	8	8	t008	1	1	t008	LONDON	CO
12593_1_16	2013	5	5	t002	1	0	t002	N WEST	HO

12593_1_17	2013	59	59	t316	1	0	t316	W MIDS	CO
12593_1_18	2013	22	22	t022	1	0	t022	W MIDS	CO
12593_1_19	2013	22	22	t032	1	0	t032	N EAST	HO
12593_1_20	2013	1	1	t127	1	0	t127	LONDON	CO
12593_1_21	2013	22	22	t032	1	0	t032	S EAST	CO
12593_1_22	2013	22	22	t032	1	0	t032	LONDON	HO
12593_1_23	2013	22	22	t032	1	0	t032	LONDON	HO
12593_1_24	2013	22	22	t020	1	0	t020	EAST	NK
12593_1_66	2013	22	22	t020	1	0	t020	EAST	CO
12593_1_26	2013	22	22	t1467	1	0	t1467	N EAST	NK
12593_1_27	2013	22	22	t032	1	0	t032	EAST	HO
12593_1_28	2013	22	22	t3861	1	0	t3861	LONDON	CO
12589_1_8	2013	22	22	t9502	1	0	t9502	S WEST	HO
12593_1_29	2013	1	1	t2279	1	0	t2279	W MIDS	HO
12593_1_30	2013	22	22	t032	1	0	t032	S EAST	HO
12593_1_68	2013	22	22	t032	1	0	t032	LONDON	CO
12593_1_69	2013	22	22	t020	1	0	t020	LONDON	HO
12589_1_9	2013	5	5	t1341	1	0	t1341	S WEST	HO
12589_1_10	2013	5	1340	t002	1	0	t002	LONDON	CO
12589_1_11	2013	1	1	t127	1	0	t127	N WEST	HO
12589_1_12	2013	22	22	t022	1	0	t022	LONDON	HO
12593_1_31	2013	unknown_CC	78	t186	1	0	t186	YORK&HUM	CO
12589_1_13	2013	22	22	t032	1	0	t032	LONDON	HO
12593_1_32	2013	5	5	t002	1	0	t002	LONDON	HO
12593_1_33	2013	22	22	t223	1	0	t223	W MIDS	CO
12589_1_17	2013	22	22	t032	1	0	t032	N EAST	NK
12593_1_72	2013	22	22	t2818	1	0	t2818	S WEST	NK
12593_1_73	2013	22	22	t5892	1	0	t5892	S EAST	HO
12589_1_19	2013	22	22	t032	1	0	t032	S WEST	NK
12593_1_35	2013	22	22	t032	1	0	t032	NK	NK
12593_1_75	2013	22	22	t032	1	0	t032	E MIDS	CO
12593_1_76	2013	22	22	t852	1	1	t852	LONDON	HO
12589_1_20	2013	5	5	t010	1	0	t010	EAST	CO
12589_1_21	2013	22	22	t032	1	0	t032	S EAST	HO
12589_1_22	2013	22	22	- (Deletions/rearrangements within the spa gene. Loss of sequence complementary to forward primer)	1	0	t032	W MIDS	CO
12589_1_50	2013	22	22	t032	1	0	t032	N WEST	HO
12593_1_37	2013	59	59	t316	1	0	t316	YORK&HUM	HO
12593_1_38	2013	22	22	t025	1	0	t025	N WEST	HO
12589_1_23	2013	22	22	t032	1	0	t032	LONDON	HO
12589_1_24	2013	unknown_CC	152	t1828	1	1	t1828	LONDON	CO
12593_1_77	2013	22	22	t022	1	0	t022	W MIDS	HO
12593_1_78	2013	30	36	t253	1	0	t253	S WEST	NK
12593_1_79	2013	30	36	t253	1	0	t253	S WEST	NK
12593_1_80	2013	22	2916	t578	1	0	t578	YORK&HUM	HO
12589_1_51	2013	22	22	t032	1	0	t032	LONDON	CO

12589_1_62	2013	30	36	t012	1	0	t012	LONDON	CO
12755_8_70	2013	22	22	t032	1	0	t032	E MIDS	CO
12593_1_40	2013	8	241	t037	1	0	t037	LONDON	CO
12593_1_41	2013	22	22	t4559	1	0	t4559	E MIDS	HO
12593_1_42	2013	22	22	t852	1	1	t852	LONDON	HO
12589_1_28	2013	22	22	t032	1	0	t032	S WEST	CO
12593_1_44	2013	22	22	t022	1	0	t022	N EAST	NK
12593_1_45	2013	22	22	t022	1	0	t022	N EAST	NK
12593_1_46	2013	30	2938	t018	1	0	t018	LONDON	CO
12589_1_63	2013	22	22	t1214	1	0	t1214	W MIDS	CO
12589_1_29	2013	22	3734	t12520	1	0	t12520	EAST	HO
12593_1_81	2013	22	3824	t022	1	0	t022	E MIDS	CO
12589_1_54	2013	30	3672	t018	1	0	t018	S EAST	HO
12593_1_82	2013	22	22	t032	1	0	t032	NK	NK
12589_1_52	2013	22	22	t032	1	0	t032	N WEST	HO
12589_1_31	2013	22	22	- (Deletions/rearrangements within the spa gene. Loss of sequence complementary to forward primer)	1	0	t032	E MIDS	CO
12589_1_32	2013	22	22	t020	1	0	t020	S EAST	HO
12593_1_83	2013	45	45	t630	1	0	t630	W MIDS	CO
12589_1_35	2013	22	22	t8473	1	0	t8473	LONDON	HO
12593_1_84	2013	22	22	t022	1	0	t022	S EAST	HO
12593_1_47	2013	22	22	t2818	1	0	t2818	S WEST	HO
12593_1_48	2013	22	22	t12254	1	0	t12254	W MIDS	NK
12593_1_49	2013	22	22	t557	1	0	t557	N EAST	HO
12593_1_50	2013	22	22	t852	1	1	t852	S WEST	CO
12593_1_51	2013	30	30	t019	1	1	t019	LONDON	CO
12593_1_52	2013	22	22	- (Deletions/rearrangements within the spa gene. Loss of sequence complementary to forward primer)	1	0	t1218	N WEST	CO
12593_1_53	2013	45	45	t026	1	0	t026	LONDON	CO
12593_1_86	2013	unknown_CC	78	t3202	1	0	t3202	YORK&HUM	HO
12593_1_87	2013	1	1	t127	1	0	t127	LONDON	HO
12589_1_55	2013	22	22	t032	1	0	t032	EAST	HO
12593_1_88	2013	22	22	t032	1	0	t032	YORK&HUM	NK
12589_1_36	2013	22	22	t6859	1	0	t6859	LONDON	NK
12593_1_89	2013	22	22	t022	1	0	t022	LONDON	CO
12593_1_90	2013	22	22	t032	1	0	t032	LONDON	CO
12593_1_91	2013	5	5	t002	1	0	t002	N WEST	HO
12589_1_53	2013	5	5	t002	1	1	t002	LONDON	HO
12589_1_37	2013	22	22	- (Deletions/rearrangements within the spa gene. Loss of sequence complementary to forward primer)	1	0	t032	W MIDS	CO
12593_1_92	2013	5	5	t002	1	0	t002	N WEST	HO
12589_1_38	2013	22	22	t032	1	0	t032	N EAST	HO
12589_1_39	2013	22	3735	t12287	1	0	t12287	LONDON	HO
12593_1_93	2013	22	22	t12293	1	0	t12293	N EAST	NK
12589_1_40	2013	22	22	t379	1	0	t379	N EAST	NK
12593_1_95	2013	22	22	t032	1	0	t032	N WEST	HO

12589_1_1	2013	30	1456	t019	1	1	t019	LONDON	CO
12589_1_2	2013	45	45	t1081	1	0	t1081	LONDON	CO
12589_1_42	2013	5	3074	t002	1	0	t002	N WEST	CO
12589_1_43	2013	22	3734	t025	1	0	t025	EAST	HO
12589_1_44	2013	22	22	t557	1	0	t557	LONDON	HO
12589_1_3	2013	1	1	t127	1	0	t127	S EAST	CO
12593_1_57	2013	5	149	t002	1	0	t002	E MIDS	CO
12589_1_45	2013	22	22	t032	1	0	t032	W MIDS	HO
12593_1_58	2013	22	22	t032	1	0	t032	LONDON	CO
12589_1_48	2013	30	3671	t018	1	0	t018	LONDON	NK
12589_1_47	2013	22	22	t1214	1	0	t1214	LONDON	CO
12589_1_46	2013	22	22	t032	1	0	t032	LONDON	HO
12589_1_4	2013	22	22	t032	1	0	t032	NK	NK
12589_1_5	2013	22	22	t032	1	0	t032	W MIDS	CO
12589_1_61	2013	59	59	t7344	1	0	t7344	YORK&HUM	CO
12593_1_59	2013	22	22	t032	1	0	t032	YORK&HUM	HO
12589_1_58	2013	8	8	t1774	1	0	t1774	S WEST	NK
12589_1_59	2013	1	1	t127	1	0	t127	N WEST	HO
12593_1_61	2013	22	22	t032	1	0	t032	S EAST	CO
12593_1_62	2013	5	5	t002	1	0	t002	N WEST	HO
12593_1_63	2013	30	36	t018	1	0	t018	LONDON	HO
12589_1_60	2013	5	5	t002	1	0	t002	S WEST	CO
12589_1_65	2013	unknown_CC	3673	t044	1	1	t044	E MIDS	HO
12589_1_66	2013	5	5	t002	1	0	t002	S EAST	CO
12589_1_67	2013	30	36	t018	1	0	t018	S WEST	HO
12589_1_68	2013	22	22	t032	1	0	t032	S WEST	HO
12589_1_69	2013	22	22	t10718	1	0	t10718	N WEST	CO
12589_1_70	2013	22	22	t032	1	0	t032	LONDON	HO
12589_1_71	2013	22	22	t032	1	0	t032	S EAST	HO
12589_1_72	2013	22	22	t020	1	0	t020	S EAST	HO
12589_1_73	2013	5	5	t002	1	0	t002	NK	NK
12589_1_74	2013	1	1	t127	1	0	t127	LONDON	CO
12755_8_71	2013	22	22	t032	1	0	t032	N WEST	CO
12755_8_72	2013	22	22	t557	1	0	t557	N EAST	NK
12589_1_77	2013	22	22	t032	1	0	t032	N EAST	CO
12589_1_78	2013	22	22	t032	1	0	t032	N EAST	CO
12589_1_80	2013	22	22	t032	1	0	t032	NK	NK
12589_1_81	2013	22	22	t032	1	0	t032	LONDON	HO
12589_1_82	2013	22	22	t032	1	0	t032	LONDON	HO
12589_1_83	2013	22	22	t12422	1	0	t12422	LONDON	HO
12589_1_84	2013	22	22	t022	1	0	t022	LONDON	NK
12589_1_85	2012	22	22	t032	1	0	t032	LONDON	HO
12589_1_86	2013	22	3737	t032	1	0	t032	LONDON	HO
12589_1_87	2013	22	22	t032	1	0	t032	YORK&HUM	CO
12589_1_88	2013	22	22	t032	1	0	t032	N WEST	NK
12593_2_77	2013	22	22	t2235	1	0	t2235	S EAST	CO

12589_1_89	2013	22	22	t2752	1	0	t2752	YORK&HUM	CO
12755_8_73	2013	22	22	t032	1	0	t032	EAST	CO
12589_1_90	2013	8	8	t008	1	0	t008	LONDON	CO
12589_1_91	2013	22	1082	t1612	1	0	t1612	S EAST	CO
12589_1_92	2013	22	3738	t2818	1	0	t2818	S WEST	NK
12589_1_94	2013	22	22	t032	1	0	t032	W MIDS	HO
12593_2_78	2013	22	22	t020	1	0	t020	EAST	CO
12589_1_95	2013	22	22	t032	1	0	t032	YORK&HUM	CO
12593_2_1	2013	22	22	t022	1	0	t022	W MIDS	CO
12593_2_2	2013	22	22	t8703	1	0	t8703	LONDON	HO
12593_2_3	2013	22	22	t032	1	0	t032	S EAST	HO
12593_2_7	2013	97	97	t6576	1	0	t6576	S EAST	CO
12593_2_8	2013	5	5	t002	1	0	t002	LONDON	CO
12593_2_9	2013	22	22	t1302	1	0	t1302	N WEST	HO
12593_2_10	2013	22	22	t022	1	0	t022	LONDON	CO
12593_2_11	2013	59	59	t7344	1	0	t7344	YORK&HUM	CO
12593_2_12	2013	22	22	t032	1	0	t032	W MIDS	CO
12593_2_13	2013	22	22	t032	1	0	t032	N WEST	NK
12593_2_14	2013	22	22	t032	1	0	t032	N WEST	HO
12593_2_15	2013	88	88	t5973	1	0	t5973	N WEST	NK
12593_2_16	2013	22	22	t12550	1	0	t12550	N WEST	CO
12593_2_17	2013	22	22	t032	1	0	t032	E MIDS	HO
12593_2_18	2013	22	22	t022	1	0	t022	EAST	HO
12593_2_20	2013	30	36	t018	1	0	t018	S EAST	CO
12593_2_21	2013	22	22	t578	1	0	t578	N WEST	HO
12593_2_22	2013	5	526	- (Deletions/rearrangements within the spa gene. Loss of sequence complementary to forward primer)	1	0	t8084	S EAST	CO
12593_2_25	2013	22	22	t032	1	0	t032	N EAST	NK
12593_2_79	2013	22	22	t032	1	0	t032	YORK&HUM	CO
12593_2_26	2013	22	22	t020	1	0	t020	S WEST	HO
12593_2_27	2013	22	3739	t032	1	0	t032	S WEST	HO
12593_2_80	2013	7	7	t12607	1	0	t12607	S WEST	CO
12593_2_28	2013	5	105	t002	1	0	t002	LONDON	CO
12593_2_74	2013	22	22	t032	1	0	t032	S EAST	HO
12593_2_29	2013	22	22	t032	1	0	t032	LONDON	HO
12593_2_30	2013	30	30	t021	1	1	t021	LONDON	CO
12593_2_31	2013	22	22	t12651	1	0	t12651	S WEST	HO
12593_2_34	2013	22	22	t020	1	0	t020	LONDON	CO
12593_2_40	2013	5	5	t002	1	0	t002	S WEST	CO
12593_2_42	2013	22	22	t022	1	0	t022	E MIDS	CO
12593_2_43	2013	22	22	t032	1	0	t032	E MIDS	HO
12593_2_37	2013	22	22	t1500	1	0	t1500	S WEST	HO
12593_2_38	2013	1	772	t657	1	1	t657	YORK&HUM	HO
12593_2_45	2013	8	8	t1774	1	0	t1774	N WEST	HO
12593_2_46	2013	22	22	t032	1	0	t032	S EAST	NK
12593_2_47	2013	22	22	t790	1	0	t790	EAST	HO

12593_2_48	2013	22	22	t032	1	0	t032	LONDON	CO
12593_2_49	2013	22	22	t032	1	0	t032	LONDON	NK
12593_2_50	2013	22	22	t025	1	0	t025	S WEST	NK
12593_2_51	2013	5	5	t1154	1	1	t1154	S EAST	CO
12593_2_52	2013	22	22	t032	1	0	t032	S WEST	HO
12593_2_53	2013	22	22	t032	1	0	t032	LONDON	HO
12593_2_54	2013	22	22	t032	1	0	t032	W MIDS	CO
12593_2_55	2013	30	30	t019	1	1	t019	S EAST	NK
12593_2_56	2013	30	36	t018	1	0	t018	YORK&HUM	HO
12593_2_81	2013	59	3740	t7344	1	0	t7344	YORK&HUM	CO
12593_2_61	2013	22	22	t032	1	0	t032	S EAST	HO
12593_2_62	2013	8	8	t008	1	0	t008	S EAST	HO
12593_2_82	2013	22	22	t020	1	0	t020	LONDON	HO
12593_2_64	2013	97	97	t12761	1	0	t12761	N WEST	HO
12593_2_57	2013	5	5	t002	1	0	t002	S WEST	NK
12593_2_58	2013	22	22	t022	1	0	t022	LONDON	CO
12593_2_59	2013	unknown_CC	361	t315	1	0	t315	LONDON	NK
12593_2_60	2013	22	22	t025	1	0	t025	S WEST	HO
12593_2_69	2013	22	22	t032	1	0	t032	LONDON	CO
12593_2_70	2013	22	22	t2892	1	0	t2892	S EAST	HO
12593_2_71	2013	22	22	t032	1	0	t032	W MIDS	CO
12593_2_83	2013	5	5	t002	1	0	t002	YORK&HUM	HO
12593_2_72	2013	22	22	t032	1	0	t032	LONDON	CO
12593_2_65	2013	22	22	t379	1	0	t379	W MIDS	HO
12593_2_84	2013	5	5	t002	1	0	t002	S WEST	CO
12593_2_66	2013	30	30	t122	1	1	t122	LONDON	NK
12593_2_67	2013	1	1	t127	1	0	t127	W MIDS	CO
12593_2_68	2013	8	241	t037	1	0	t037	LONDON	HO
12593_2_91	2013	30	36	t018	1	0	t018	LONDON	NK
12593_2_92	2013	22	22	t032	1	0	t032	LONDON	HO
12593_2_93	2013	22	22	t032	1	0	t032	E MIDS	CO
12593_2_95	2013	1	1	t127	1	0	t127	S EAST	HO
12673_8_44	2013	8	8	t008	1	1	t008	W MIDS	NK
12673_8_78	2013	22	3757	t032	1	0	t032	LONDON	CO
12673_8_60	2013	8	8	t008	1	1	t008	S EAST	HO
12673_8_61	2013	unknown_CC	361	t315	1	0	t315	LONDON	CO
12673_8_70	2013	8	241	t037	1	0	t037	LONDON	NK
12673_8_71	2013	22	22	t020	1	0	t020	S WEST	HO
12673_8_77	2013	8	8	t008	1	0	t008	S EAST	CO
12673_8_62	2013	8	8	t008	1	0	t008	YORK&HUM	CO
12673_8_63	2013	1	1	t127	1	0	t127	S WEST	CO
12673_8_66	2013	5	5	t002	1	0	t002	S WEST	NK
12673_8_55	2013	5	149	t002	1	0	t002	LONDON	NK
12673_8_56	2013	22	22	t032	1	0	t032	S EAST	NK
12673_8_68	2013	5	5	t002	1	0	t002	E MIDS	HO
12673_8_69	2013	22	22	t032	1	0	t032	E MIDS	HO

12673_8_76	2013	22	22	t005	1	1	t005	S EAST	CO
12673_8_51	2013	1	1	t127	1	0	t127	S WEST	HO
12673_8_52	2013	22	22	t032	1	0	t032	YORK&HUM	CO
12673_8_79	2013	22	22	t032	1	0	t032	N WEST	NK
12673_8_80	2013	88	88	t729	1	0	t729	S EAST	CO
12673_8_81	2013	5	5	t002	1	0	t002	S EAST	NK
12673_8_54	2013	1	1	t127	1	0	t127	S WEST	HO
12673_8_57	2013	22	22	t032	1	0	t032	E MIDS	HO
12673_8_58	2013	5	5	t002	1	0	t002	EAST	HO
12673_8_59	2013	45	3846	t015	1	0	t015	W MIDS	HO
12593_2_85	2013	22	22	t022	1	0	t022	N EAST	NK
12673_8_45	2013	22	22	t032	1	0	t032	E MIDS	HO
12673_8_46	2013	8	241	t030	1	0	t030	N WEST	HO
12673_8_48	2013	22	22	t032	1	0	t032	N WEST	NK
12673_8_47	2013	8	8	t032	1	0	t032	S EAST	HO
12673_8_50	2013	30	36	t018	1	0	t018	S EAST	CO
12673_8_53	2013	22	22	t005	1	1	t005	S EAST	HO
12673_8_72	2013	22	22	t718	1	0	t718	YORK&HUM	HO
12673_8_73	2013	22	22	t032	1	0	t032	N WEST	HO
12673_8_74	2013	97	97	t267	1	0	t267	LONDON	HO
12593_2_87	2013	22	22	t492	1	0	t492	S EAST	HO
12593_2_88	2012	22	22	t032	1	0	t032	YORK&HUM	CO
12593_2_89	2013	22	22	t1802	1	0	t1802	YORK&HUM	CO
12593_2_90	2013	30	36	t012	1	0	t012	YORK&HUM	HO
12593_2_86	2013	22	22	t022	1	0	t022	YORK&HUM	CO
